# *In-Silico* discovery of Pediatric Acute-Myeloid-Leukemia (pAML) causing druggable molecular signatures highlighting their pathogenetic processes and therapeutic agents through single-cell RNA-Seq profile analysis

**DOI:** 10.1371/journal.pone.0335410

**Published:** 2025-10-31

**Authors:** Md. Foysal Ahmed, Md. Al Noman, Md. Feroj Ahmed, Md. Abdul Latif, Md. Al Amin Pappu, Md. Shariful Islam, Md. Sanoar Hossain, Md. Bayazid Hossen, Md. Fahim Faysal, Md. Mehedi Hasan, Md. Nurul Haque Mollah

**Affiliations:** 1 Bioinformatics Lab (Dry), Department of Statistics, University of Rajshahi, Rajshahi, Bangladesh; 2 Institute of Bangladesh Studies (IBS), University of Rajshahi, Rajshahi, Bangladesh; 3 Department of Agricultural and Applied Statistics, Bangladesh Agricultural University, Mymensingh, Bangladesh; 4 Department of Computer Science and Engineering (CSE), Rajshahi University of Engineering and Technology (RUET), Rajshahi, Bangladesh; 5 Division of Biotechnology and Molecular Medicine, Department of Pathobiological Science, School of Veterinary Medicine, Louisiana State University, Baton Rouge, Louisiana, United States of America; Tarbiat Modares University, IRAN, ISLAMIC REPUBLIC OF

## Abstract

Pediatric acute-myeloid-leukemia (pAML) is an aggressive malignancy and the second most common blood cancer in children. In spite of significant advances in the frontline therapeutic approaches, approximately 50% of pAML patients show poor prognosis and relapse. Though drugs show positive response against the cancer cells initially, however, it becomes resistant in the long run of treatment, requiring the use of alternative drugs. Therefore, this study aimed to discover pAML-causing druggable molecular signatures highlighting their pathogenetic processes and alternative therapeutic agents. To address these issues, at first, we performed an integrated single-cell RNA sequencing (scRNA-seq) profile analysis of two datasets with accession IDs GSE154109 and GSE235923, which revealed 6 pAML-related key cell types (Erythroid cells, GdT-cells, Naive B-cells, Naive CD4 T-cells, Non-Classical Monocytes, and T-regs) and 198 common differentially expressed genes (cDEGs) between pAML and healthy groups. The protein-protein interaction (PPI) analysis yielded top-ranked eight cDEGs (JUN, MDM2, FOS, SOD2, FBXW7, CHD3, MCL1, and MAP2K1) as common key genes (cKGs) across the key cell types. Disease-cKGs enrichment analysis further confirmed the relevance of these genes to pAML and other leukemic diseases. Regulatory network analysis identified top four transcription factors (FOXC1, GATA2, RELA, and TP53) and three microRNAs (hsa-let-7a-5p, hsa-let-7e-5p, hsa-miR-15a-5p) that regulate these cKGs. Gene ontology (GO) terms and Kyoto Encyclopedia of Genes and Genomes (KEGG) pathway enrichment analysis results reflected their potential roles in pAML pathogenesis. Pathway perturbation analysis through gene-set enrichment analysis (GSEA) tool identified significantly perturbed pathways, highlighting how they are altered in pAML environment and how the cKGs are linked in the process. Subsequently, three potential therapeutic candidates (IRINOTECAN HYDROCHLORIDE, IMATINIB and IBRUTINIB) were disclosed through an integrative strategy combining molecular docking, drug-likeness, ADME/T, and DFT analyses. Molecular dynamics (MD) simulation studies for the top three drug-target complexes indicated the stability of complexes. Thus, the findings potentially offer valuable insights for pAML pathogenesis and effective therapeutic candidates for pAML patients.

## 1. Introduction

**Fig 1:**
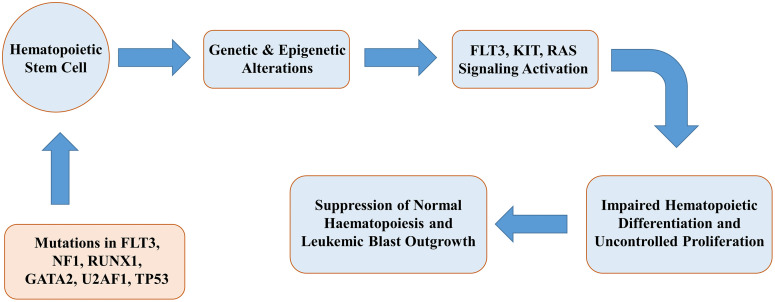
A schematic diagram about the molecular mechanisms of pAML development.

Pediatric acute myeloid leukemia (pAML) is a form of blood cancer that arises when immature myeloid cells in the bone marrow acquire self-renewal ability, fail to differentiate properly, and proliferate uncontrollably [1]. This aggressive disease disrupts normal blood cell development and threatens survival if left untreated [2]. Briefly, it is a serious blood cancer that ranks as the second most frequent in children, representing approximately 20–25% of all pediatric leukemia cases [3]. It affects about seven children per million, independent of geographic region [4]. While about 70% of young patients now survive the disease, this also means that nearly one-third still don’t overcome it [5]. In high-income countries, the 5-year survival rate for pAML has risen to nearly 75% due to better diagnostics, treatments, supportive care, and salvage therapies like stem cell transplantation. However, low- and middle-income countries have not seen equal progress [6]. Besides, despite notable progress in frontline therapies, nearly 50% of pAML patients experience poor prognosis and relapse [7]. Although initial responses to treatment are often favorable, many patients eventually develop resistance [8], necessitating the search for alternative therapeutic options. In healthy children, bone marrow function relies on hematopoietic stem cells (HSCs) differentiating into myeloid and lymphoid stem cells, which eventually generate mature blood cells [9]. However, in pAML, this orderly differentiation process is disrupted. pAML is initiated by genetic and epigenetic alterations in hematopoietic stem cells. These aberrations disrupt critical signalling pathways, which leads to impaired hematopoietic differentiation and uncontrolled proliferation of myeloid progenitors. Consequently, this results in the suppression of normal haematopoiesis and immature cells, i.e., leukemic blasts multiply excessively and overcrowd the bone marrow, reducing the space available for normal blood cell production, including healthy white blood cells, red blood cells, and platelets within the bone marrow microenvironment [10,11]. Therefore, it is essential to identify pAML-related genes dysregulated due to mutations or epigenetic alterations, for exploring candidate drugs. A schematic diagram about the mechanism of pAML development is given in **Fig 1**.

In some earlier studies, bulk microarray gene expressions [12,13] and RNA-Seq profiles [14] were used to identify pAML-causing genes, but their findings were not so consistent. It should be mentioned here that single-cell RNA sequencing (scRNA-seq) profile analysis shows better performance than bulk microarray and RNA-seq methods in identifying disease-related genes because it provides high-resolution, cell-specific data that can reveal cell heterogeneity, rare cell populations, and gene expression patterns masked in bulk tissue analyses [15–17]. Therefore, in this study, we considered scRNA-seq profile datasets to identify pAML-causing genes more accurately highlighting their pathogenetic processes and candidate drugs, since, so far, no researchers rigorously explored pAML-causing genes through scRNA-seq profile analysis.

## 2. Materials and methods

This section outlines the data sources and descriptions, preprocessing steps, computational tools, methods employed to identify pAML-causing key genes from scRNA-seq data, their regulators and pathogenetic processes, and procedures to explore candidate drug molecules as introduced in the following sub-sections 2.1–2.12.

### 2.1. Data source and descriptions

In this study, the scRNA-seq count datasets were obtained from the Gene Expression Omnibus (GEO) platform of the National Center for Biotechnology Information (NCBI) database with accession numbers GSE154109 [18] and GSE235923 [19]. Dataset GSE154109 consisted of 4 control samples, 8 pAML samples, and 7 pALL samples, while the dataset GSE235923 consisted of 19 pAML before treatment, 10 pAML after treatment, and 2 pAML(relapse) samples. The dataset with accession ID GSE154109 was analyzed to characterize bone marrow T cells and investigating their impact on leukemia risk classification [18]. The other dataset with accession ID GSE235923 was analyzed to investigate (i) the differences in the bone marrow environment between relapse and remission after treatment [19], and (ii) the role of ARMH1 gene in pAML and relapsed patients [20], but none of these studies identify key genes (KGs) associated with pAML. Therefore, we combined these two datasets to identify pAML-causing KGs. The detail description of these datasets was given in S1 Table.

### 2.2. Data processing and integration

The scRNA-seq count data were processed using Scanpy [21] in Python for standard quality control, normalization, and feature selection. To identify and remove doublets, we applied SOLO [22], ensuring only singlet cells were retained. Then, we filtered out low quality cells and genes. Afterward, the data were normalized and log-transformed to stabilize variance in gene expression values. We integrated the data using the scVI package, which employs a Variational Autoencoder deep learning approach to minimize unwanted variation, correct batch effects, reduce dimensionality, and generate a low-dimensional representation for further analysis [23]. Then we compared its results with the results of alternative Harmony integration [24]. We then identified 5000 highly variable genes through scVI integrated data due to its better performance and retrieved approximately 55,503 high-quality single-cell transcriptomes for downstream analysis. For more detailed description, please see S1 File.

### 2.3. Cell clustering

We constructed a cellular neighborhood graph based on gene expression similarities and visualized it in 2D using Uniform Manifold Approximation and Projection (UMAP) [25]. We then employed the Leiden [26] algorithm which successfully partitioned the cellular neighborhood graph into 21 distinct clusters based on differences and similarities of gene expression patterns. Differential expression analysis was conducted to identify the top 20 marker genes for each cluster. Finally, cell type annotation was performed by integrating reference databases, including the Human Protein Atlas [27], CellMarker 2.0 [28], and PanglaoDB [29], ensuring accurate and biologically meaningful annotation. For more detailed description, please see S2 File.

### 2.4. Identification of cluster-specific marker genes and corresponding cell types

To identify cell-type-specific markers, differential expression pattern analysis was conducted across all clusters using the ‘sc.get.rank_genes_groups_df’ function. The Wilcoxon test was employed, and genes were considered differentially expressed (DEGs) if they met the significance criteria of *p*-value < 0.05 and |logFC| > 1. To assess marker gene specificity, a cell-type specificity score was used to quantify the uniqueness of marker genes to particular cell populations [30]. For more detailed description, please see S3 File.

### 2.5. Cell-cell communication analysis for exploring pAML-related cell types

Cell-cell communication (CCC) analysis was performed using LIANA+ and Tensor-cell2cell computational frameworks to identify key cell types. LIANA+ [31] integrates multiple ligand-receptor databases and analytical methods to predict ligand-receptor interactions, while Tensor-cell2cell [31] organizes CCC scores into a four-dimensional (4D) tensor framework, enabling the identification of coordinated signaling patterns across multiple samples. Spearman correlation was used to find monotonic, potentially non-linear associations between disease and healthy conditions in each factor. By combining these tools, we systematically analyzed ligand-receptor interactions from context-specific cell-cell communication networks and thus identified the key cell types driving pAML [32].

### 2.6. Identification of cell type specific common DEGs (cDEGs)

Differentially expressed genes (DEGs) for each annotated cell type were identified. We conducted differential expression analysis for scRNA-seq data using the t-test with the ‘de.test.t_test’ function from the diffxpy package in Python [33]. Significant differentially expressed genes (DEGs) were defined using thresholds of p-value < 0.05 and |logFC| > 1. Then, cDEGs across key cell types were obtained and visualized using Jvenn [34]. For more detailed description, please see S4 File.

### 2.7. Identification of pAML-causing common key genes (cKGs) from cDEGs

A protein-protein interaction (PPI) network was constructed using the STRING (version 12.0) [35] database to identify pAML-causing common key genes (cKGs). We used Cytoscape (version 3.8.2) [36] for network visualization, where nodes represented proteins and edges indicated their interactions. A Cytoscape plugin: CytoHubba [37] was applied for network analysis, using five topological measures: CLOSENESS, DEGREE, EPC, MNC, and RADIALITY to rank network nodes based on shortest-path calculations. cKGs were identified by focusing on the top-ranked proteins after comprehensive network evaluation.

### 2.8. Verification of association between cKGs and pAML using independent database

We performed cKGs-disease enrichment analysis using the DisGeNET [38] database via the Enrichr [39] platform, allowing us to investigate their associations with different leukemic conditions, including pAML.

### 2.9. Disclosing pathogenetic processes of cKGs

To disclose pathogenetic processes of cKGs, we performed cKGs regulatory network analysis with transcription factors (TFs) proteins and micro-RNAs (miRNAs), and enrichment analysis with gene ontology (GO) terms and KEGG-pathways as introduced in sub-sections 2.9.1 and 2.9.2.

#### 2.9.1. cKGs regulatory network analysis.

Interaction network analyses were conducted using the NetworkAnalyst 3.0 [40] platform to pinpoint the key regulatory transcription factors (TFs) and micro-RNAs (miRNAs) of the cKGs in conjunction with the JASPAR [41] and TarBase [42] databases for TFs-cKGs and miRNAs-cKGs interactions, respectively. Cytoscape (version 3.8.2) was used for network visualization, and core regulatory elements were identified using Betweenness [43] and Degree [44] topological analyses.

#### 2.9.2. cKGs-set enrichment analysis with GO-terms and KEGG-pathways.

We performed cKGs-set enrichment analysis with gene ontology (GO) terms using DAVID (v2024q4) [45] web tool to disclose key biological processes (BPs), molecular functions (MFs), and cellular components (CCs) associated with pAML. In order to disclose pAML-related signaling pathways, we performed cKGs-set enrichment analysis with the Kyoto Encyclopedia of Genes and Genomes (KEGG) pathways using the same web tool, DAVID [45].

#### 2.9.3. Pathway perturbation analysis using GSEA.

Pathway perturbation analysis through Gene-Set Enrichment Analysis (GSEA) identifies significantly enriched perturbed pathways associated with pAML [46,47]. The cDEGs associated with pAML were ranked by their log_2_FC values, and enrichment analysis was performed through GSEA. It employs a permutation-based approach to assess the significance of pathway activation or suppression. Permutation-based testing in GSEA provides a distribution-free way to evaluate whether observed enrichment scores are higher than expected by chance. This approach is especially suitable for pathway-level analysis because it accounts for gene-gene correlations and avoids strong parametric assumptions.The threshold *p*-values <0.05 were used to curate the enriched perturbed pathways significantly. The analysis was conducted using GSEApy (v1.1.9) package in Python [48] using the ‘KEGG 2021 Human’ database with 100 permutations and four parallel processes to assess statistical significance. Results were generated with a fixed random seed of 42 to ensure reproducibility.

### 2.10. Exploring cKGs-guided drug molecules against pAML

To explore potential repurposable drug molecules, we conducted molecular docking analysis. Drug target receptors were considered to be cKGs-mediated proteins and associated TFs protein. We sourced 284 candidate drug agents from the literature and two databases: protein-guided drugs from DGIdb [49] and disease-guided drugs from DrugBank [50] (S8 Table). The structures of target proteins were obtained from the Protein Data Bank (PDB) [51] and AlphaFold [52], whereas the PubChem [53] database provided the structures of 214 meta-drug compounds. Receptor proteins were prepared through charge addition and energy minimization before conducting molecular docking analysis with AutoDock Vina (v1.2.x) [54]. Finally, we utilized BIOVIA Discovery Studio 2021 (v21.1.0.20298) [55] to thoroughly analyze and visualize the molecular docking results of 196 meta-drugs, facilitating detailed interaction modeling. For more detailed description, please see S5 File.

### 2.11. *In-Silico* validation of candidate drugs

To computationally validate the identified candidate drugs, we carried out drug-likeness evaluation along with ADMET (Absorption, Distribution, Metabolism, Excretion, and Toxicity) and DFT (Density Functional Theory) analyses to assess their pharmacokinetic behavior, safety profile, and molecular stability.

#### 2.11.1. Drug-likeness and ADME/T analysis.

To analyze the structural and chemical properties of the top 10 drug molecules, we assessed drug-likeness and ADMET (Absorption, Distribution, Metabolism, Excretion, and Toxicity) profiles. SwissADME [56] and pkCSM [57] was used to evaluate compliance with Lipinski’s Rule of Five [58], as well as predicted ADME/T properties using Simplified Molecular Input Line Entry Specification (SMILES)-encoded optimized structures. Compounds meeting both Lipinski and ADMET criteria were shortlisted as potential drug candidates. Protein-Ligand Interaction Profiler (PLIP) [59] analyzed docked complexes, and drug-receptor interactions were further examined using BIOVIA Discovery Studio 2021.

#### 2.11.2. Cross-validation of the proposed drugs with the independent receptors.

To further evaluate the therapeutic potential of the proposed drug candidates for pAML therapy, we assessed their binding interactions with 27 different independent receptors previously detected as pAML-causing key genes (KGs) or hub-genes (HubGs) in different published articles (S10 Table). We conducted molecular docking analyses to determine the binding affinities between these key receptors and our suggested compounds to provide additional validation for the proposed therapeutic candidates.

#### 2.11.3. Validation of docking specificity using negative control.

To investigate the docking specificity, we considered 50 decoy molecules as negative controls for each of the proposed drug molecules that are structurally related but assumed to be inactive counterparts. The decoy molecules were generated from the Directory of Useful Decoys, Enhanced (DUDE) database, which provides high-quality decoys for benchmarking docking studies [60]. Then we performed docking analysis between the proposed target proteins and the decoy ligands to confirm that the original binding affinities are stronger than the binding affinities with the decoy molecules [61].

#### 2.11.4. Density functional theory (DFT) analysis.

Density Functional Theory (DFT) analysis was performed to assess the physicochemical properties (structural, functional, and quantum chemical) of the selected drug molecules identified through molecular docking, drug-likeness, and ADMET profile analyses. Calculations were conducted using Gaussian-09 (version 9.5) [62] with the B3LYP method [63] and 6-311G basis set [64] to determine thermodynamic energies, electronic properties, and frontier molecular orbital (FMO) energies in the gaseous state. The energy of the highest occupied molecular orbital (HOMO), lowest unoccupied molecular orbital (LUMO), and energy gap (ΔE) between them were computed using Gaussian-09 and visualized with GaussView-6 [65] for the drug molecules. The mathematical definitions of the required parameters are given in S6 File.

### 2.12. Molecular dynamics (MD) simulation

Molecular dynamics (MD) simulations of the top protein–ligand complexes were conducted using YASARA software [66] with the AMBER14 force field to assess their dynamic behavior. Three complexes with the best docking scores were selected and simulated for 100 ns under certain physiological conditions. Trajectories were saved every 100 ps for analysis with YASARA macros [67] and SciDAVis (http://scidavis.sourceforge.net/). Binding free energies (ΔGbind) were calculated every 100 ps using the MM-PBSA method in YASARA to evaluate interaction stability [68]. For more detailed description, please see S7 File.

## 3. Results

This section presents an integrated overview of the pre-processing outcomes, cell clustering with marker gene identification, cell type annotation, key genes discovery, their regulators and biological functions in pAML, and the discovery of candidate drug molecules as described in the following sub-sections 3.1–3.12.

### 3.1. Obtaining high-quality cells by preprocessing of scRNA-seq count data

**Fig 2:**
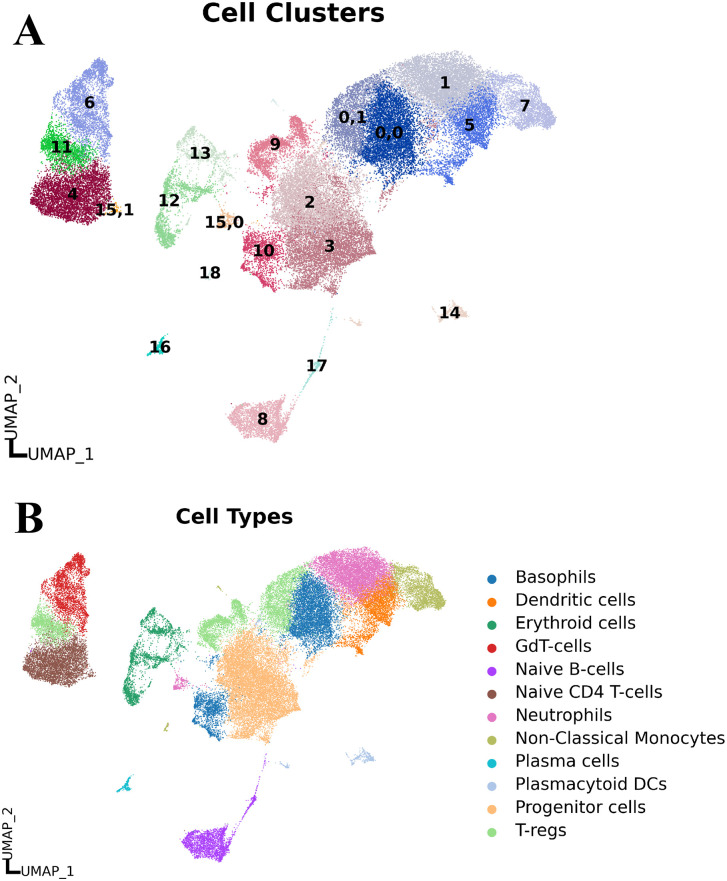
UMAP plot of cell clusters. **(A)** Displays distinct communities within the cell clusters. Each dot represents a cell, and each color represents a distinct community. **(B)** shows annotated cell types to the clusters. Here, each dot represents a cell, and each color represents a distinct cell type.

From the raw dataset, we initially identified 92,896 cells. After filtering and removing doublets, we got 82,855 cells. Following the stringent quality control measures as described previously, we got 55,503 high-quality cells. We observed clear sample-specific clustering (S1A Fig) before correcting batch effects, indicating strong batch effects in our data. Implementation of scVI [23] for batch correction resulted in a more even cellular distribution (S1B Fig), demonstrating effective removal of technical variability between samples. Then we compared the results of scVI-based integrated with the results of Harmony-based integrated data clustering, normalized mutual information (NMI) and Graph connectivity scores. We observed that scVI-based integrated data perform better than Harmony-based integrated data in the case of cell type clustering (Fig 2B and S2 Fig). The normalized mutual information (NMI) score for scVI and Harmony were 0.91 and 0.54, respectively, which indicates better clustering by scVI approach. Graph connectivity scores with scVI and Harmony approaches were 0.997 and 0.988, respectively, reflecting stronger preservation of biological structure by scVI-integration.

### 3.2. Identification of cell clusters and cluster-specific marker genes

We identified 21 distinct cell clusters (**Fig 2A**) and determined their identities by selecting the top 20 highly expressed genes per cluster based on the highest log fold change (logFC) values (S2 Table). Marker genes were validated using CellMarker 2.0 [28], Human Protein Atlas [27], and PanglaoDB [29] to ensure cell type specificity. We specified a cell type to a cluster by following the Khan et al., 2022 classification criteria (≥50% marker gene match per cell type) [69].

### 3.3. Identification of cell types

From the 21 identified cell clusters, 12 distinct cell types were characterized, including Basophils, Dendritic cells, Erythroid cells, Gamma delta T cells (GdT-cells), Neutrophils, Plasma cells, Progenitor cells, T regulatory cells (T-regs), Naive B cells, Naive CD4 T cells, Plasmacytoid Dendritic cells (Plasmacytoid DCs), and Non-Classical Monocytes. (**Fig 2B**).

The top marker genes identified for each cell-type included: Basophils (SOCS2), Dendritic cells (CEBPD), Erythroid cells (SNCA, BLVRB), GdT-cells (GZMA, CCL5), Neutrophils (LYZ), Plasma cells (MZB1, FKBP11), Progenitor cells (EGFL7, PRSS57), T-regs (CENPM), Naive B cells (MS4A1, BANK1), Naive CD4 T-cells (LEF1, TCF7), Non-Classical Monocytes (FCGR3A, CDKN1C), and Plasmacytoid DCs (PLD4, TCF4) (S3 Table). The matrix plot graphs in S3 Fig visualized the key marker genes for each cell type.

### 3.4. Cell-cell communication analysis for exploring pAML-causing key cell types

Context specific cell-cell communication (CCC) analysis revealed nine key factors distinguishing healthy and pAML cellular interactions. The relationship between factor loadings and disease conditions was evaluated using Spearman correlation coefficients. Among all factors analyzed, Factor 4 emerged as particularly important with a Spearman correlation coefficient of 0.58, showing the strongest correlation between healthy and pAML conditions in **Table 1**. The loading pattern of Factor 4 reveals how cellular communication networks are altered during disease development. Biologically, this means that the set of ligand–receptor interactions and cell–cell communication patterns captured by factor 4 are more prominently engaged in pAML.

**Table 1:** Spearman correlation and p-values of each factor between Healthy and pAML samples.

Factors	Spearman Coefficient	*p*-value
Factor 1	0.05	0.77
Factor 2	−0.41	0.02
Factor 3	−0.19	0.30
Factor 4 *	0.58	0.0006
Factor 5	−0.40	0.03
Factor 6	0.04	0.82
Factor 7	−0.19	0.30
Factor 8	0.09	0.65
Factor 9	−0.06	0.73

Asterisks (*) indicate the most significant factors.

**Fig 3:**
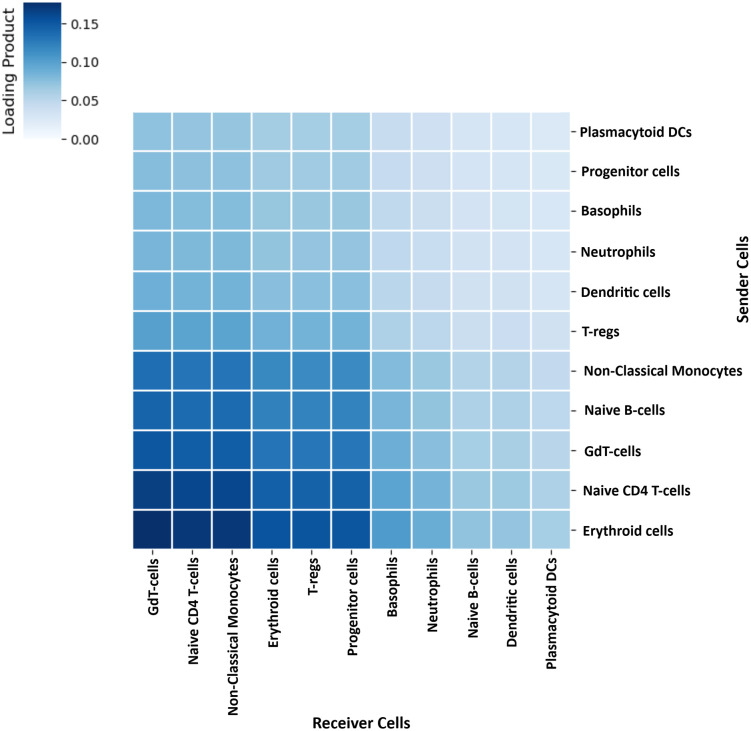
Context-specific cell-cell communication network. Rows represent sender cell types, and columns represent receiver cell types. Color intensity indicates communication strength, with darker shades reflecting stronger interactions.

The dominant sender cells were Erythroid cells, Naive CD4 T-cells, GdT-cells, Naive B-cells, and Non-Classical Monocytes. Notably, GdT-cells, Naive CD4 T-cells, Non-Classical Monocytes, Erythroid cells, and T-regs acted as the key receiver cells (Fig 3 and S4 Table). From these findings, we obtained 6 key cell types: Erythroid cells, GdT-cells, Naive B-cells, Naive CD4 T-cells, Non-Classical Monocytes, and T-regs for pAML progression.

### 3.5. Identification of key cell-type specific common differentially expressed genes (cDEGs)

We analyzed DEGs of 6 key cell types identified from context-specific cell-cell communication. Upregulated and downregulated DEGs for these cell types were obtained through t-tests with a threshold of |logFC| > 1 and *p*-value < 0.05. By intersecting the DEGs across these key cell types, we identified 198 common differentially expressed genes (cDEGs), where 154 genes were upregulated and 44 were downregulated (S4 Fig and S5 Table).

### 3.6. Identification of cKGs from cDEGs

**Fig 4:**
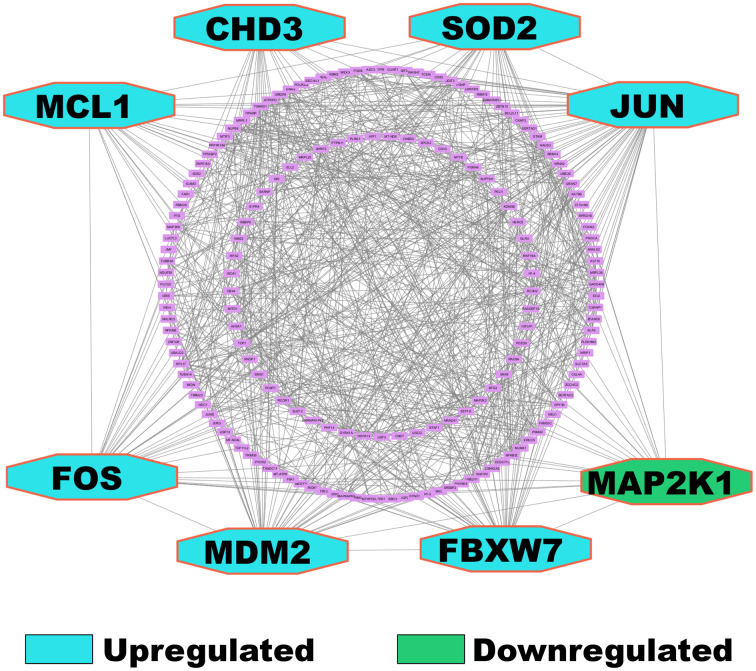
Protein-protein interaction (PPI) network of cDEGs to identify cKGs, where the cyan octagons represent upregulated cKGs, and the green octagon represents downregulated cKG.

We constructed a protein-protein interaction (PPI) network to analyze the relationships between the cDEGs using the STRING [70] database. The resulting network encompassed 177 nodes representing individual genes and 811 edges showing their interactions. We evaluated five different network topology measures: CLOSENESS, DEGREE, EPC, MNC, and RADIALITY to identify the cKGs (S6 Table). This led to the identification of eight cKGs: JUN, MDM2, FOS, SOD2, FBXW7, CHD3, MCL1, and MAP2K1. Among these, MAP2K1 is downregulated, and the remaining seven are upregulated (**Fig 4**).

### 3.7. Verification of association between cKGs and pAML using an independent database

The top 25 diseases most closely related to cKGs are listed in S7 Table, with statistical significance set at *p*-value < 0.05. Analysis of disease-cKGs interactions confirmed a strong association between cKGs and pAML, along with links to various leukemic diseases.

### 3.8. cKGs regulatory network analysis

**Fig 5:**
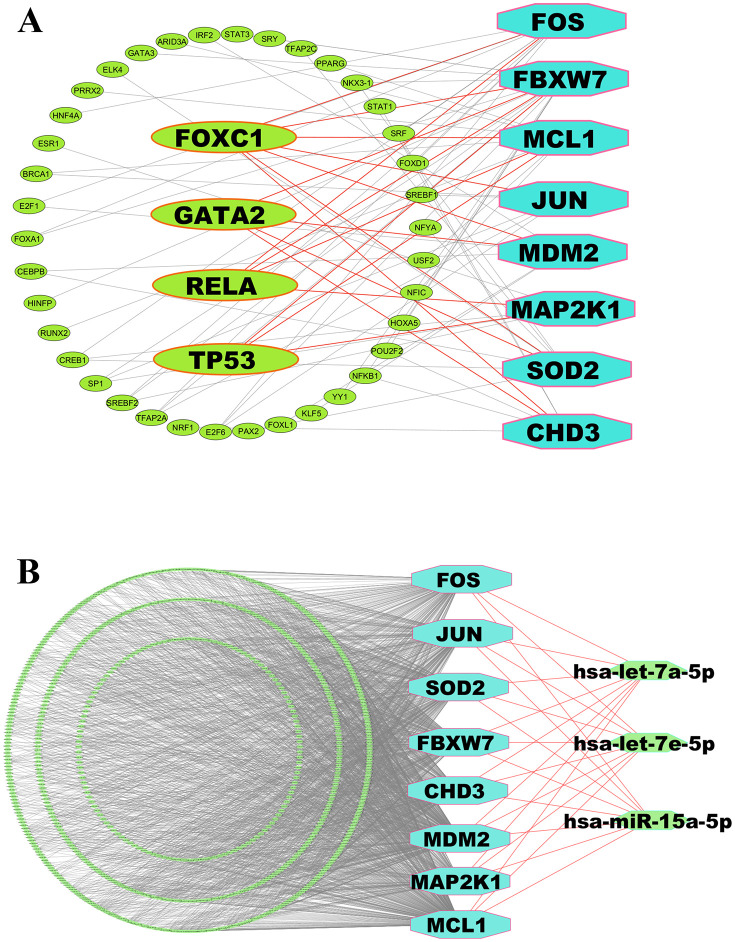
Regulatory network of cKGs. (A) displays cKGs-TFs interaction network, where cyan octagons represent cKGs and larger green ellipses represent top TFs. (B) displays cKGs-miRNAs interaction network, where cyan octagons represent cKGs and larger green hexagons represent top miRNAs.

The transcriptional and post-transcriptional regulators of cKGs were determined by analyzing their interactions with TFs and miRNAs. Based on degree (≥3) and betweenness (≥50), the top 4 transcriptional regulators (FOXC1, GATA2, RELA, and TP53) were identified (**Fig 5A**). Similarly, for post-transcriptional regulation, the top 3 miRNAs (hsa-let-7a-5p, hsa-let-7e-5p, and hsa-miR-15a-5p) were identified using degree (≥8) and betweenness (≥660) (**Fig 5B**).

#### 3.9.1. cKGs-set enrichment analysis with GO-terms and KEGG-pathways.

In this study, GO and KEGG pathway enrichment analyses were performed on the eight cKGs using the DAVID database [57] to investigate the underlying pathogenic mechanisms of pAML. The top-enriched biological processes (BPs), cellular components (CCs), molecular functions (MFs), and KEGG pathways are summarized in **Table 2**.

**Table 2:** Significantly (*p*-value<0.05) enriched GO terms and KEGG pathways.

Biological Process			
GO and KEGG ID	Description	P-Value	Associated KGs
GO:1903378	positive regulation of oxidative stress-induced neuron intrinsic apoptotic signaling pathway	0.0014	FBXW7, MCL1
GO: 0045893	positive regulation of DNA-templated	0.0015	MAP2K1, JUN, FOS, CHD3
GO: 0009410	response to xenobiotic stimulus	0.0035	MDM2, FOS, SOD2
GO: 0048545	response to steroid hormone	0.0079	JUN, MDM2
**Cellular Component**			
GO:0035976	transcription factor AP-1 complex	0.0020	JUN, FOS
GO:0005634	nucleus	0.0036	MAP2K1, JUN, FBXW7, MDM2, FOS, CHD3, MCL1
GO:0005654	nucleoplasm	0.0040	JUN, FBXW7, MDM2, FOS, CHD3, MCL1
GO:0005739	mitochondrion	0.0154	MAP2K1, FBXW7, SOD2, MCL1
**Molecular Function**			
GO:0042802	identical protein binding	0.0020	JUN, FBXW7, MDM2, FOS, SOD2
GO:0000976	transcription cis-regulatory region binding	0.0032	JUN, FOS, CHD3
GO:0031625	ubiquitin protein ligase binding	0.0052	JUN, FBXW7, MDM2
GO:0019899	enzyme binding	0.0074	JUN, MDM2, SOD2
**KEGG Pathway**			
hsa01522	Endocrine resistance	4.58E-05	MAP2K1, JUN, MDM2, FOS
hsa04210	Apoptosis	1.18E-04	MAP2K1, JUN, FOS, MCL1
hsa05208	Chemical carcinogenesis – reactive oxygen species	5.37E-04	MAP2K1, JUN, FOS, SOD2

#### 3.9.2. Pathway perturbation analysis using GSEA.

The results of the pathway perturbation analysis through GSEA were displayed in Table 3. From this Table, it is seen that the IL-17 signaling pathway is strongly activated in pAML, with a normalized enrichment score (NES) of 1.74 and a *p*-value of 0, identifying FOS as an associated core gene. The osteoclast differentiation pathway was also activated (NES = 1.52, p = 0.04) with FOS and JUN as key contributing genes. In contrast, the cellular senescence pathway was suppressed in pAML (NES = −1.53, p = 0.036), with MAP2K1 highlighted as an associated core gene. These findings suggest activation of two pathways alongside suppression of senescence mechanisms in pAML pathology.

**Table 3:** Activated signaling pathways in pAML based on GSEA analysis.

Pathways	NES	*p*-value	Interpretation	Associated cKGs
IL-17 Signaling Pathway	1.737983	0	Activated in pAML	FOS
Osteoclast Differentiation	1.515297	0.04	Activated in pAML	FOS, JUN
Cellular Senescence	−1.52694	0.035714	suppressed in pAML	MAP2K1

### 3.10. Exploring cKGs-guided drug molecules against pAML

**Fig 6:**
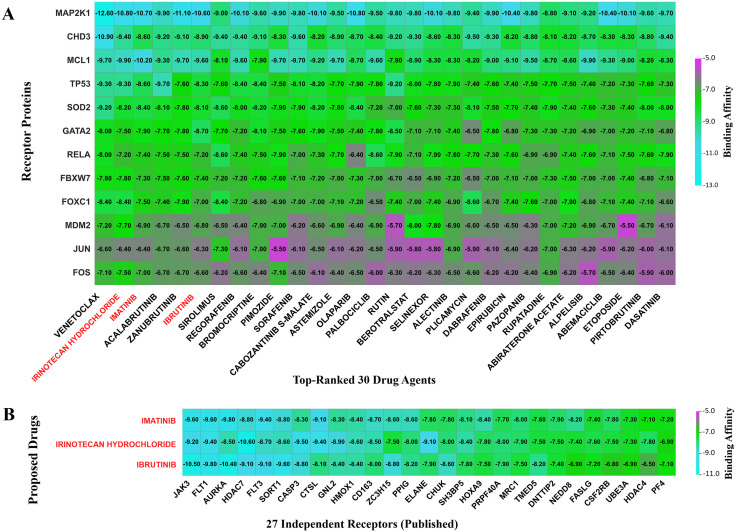
Molecular docking analysis of candidate drug agents with receptor proteins. **(A)** Heatmap displaying binding affinity scores between receptor proteins (rows) and drug agents (columns). Three candidate drugs highlighted in red demonstrate superior binding affinities with receptor proteins and were proposed as promising therapeutic candidates following additional validation studies. **(B)** Heatmap displaying binding affinity scores between the proposed drugs (rows) and receptor proteins (columns) previously published as KGs or HGs.

In this study, we selected 12 drug target receptors, consisting of eight cKGs (JUN, MDM2, FOS, SOD2, FBXW7, CHD3, MCL1, and MAP2K1) and four TFs (FOXC1, GATA2, RELA, TP53). We obtained the 3D structures of FBXW7, FOS, GATA2, MAP2K1, MCL1, MDM2, SOD2, and TP53 from the Protein Data Bank and JUN, CHD3, FOXC1, RELA from AlphaFold, using their corresponding identifiers: 5ibk, 1s9k, 5o9b, 3eqc, 2kbw, 2hdp, 1zte, 1kzy, P05412, Q12873, Q12948, and Q04206 respectively. Molecular docking was conducted to evaluate the binding affinity scores (BASs) between target receptors and candidate drug molecules (S9 Table). Receptors and drug candidates were ranked based on the row and column averages of the BASs matrix. The heatmap illustrates the top 30 drug molecules with the highest binding affinities to target receptors (**Fig 6A**).

### 3.11. *In-silico* validation of candidate drugs

#### 3.11.1. Drug likeness profile ADME/T analysis.

The drug-likeness of the top 10 candidate drugs with the highest average BAS with the target receptors were assessed based on Lipinski’s Rule of Five (ROF), which encompasses criteria for molecular weight (≤500), log P (≤5), hydrogen bond donors (≤5), and hydrogen bond acceptors (≤10). Allowing only a single violation, it was found that certain compounds met the drug-likeness criteria. (**Table 4**).

**Table 4:** Drug-likeness profile of the top 10 drugs.

Molecular properties								Lipinski Rule	
Compounds	Molecular weight	Log P	Water solubility (log mol/L)	H-bond Acceptor (HBA)	H-bond donor (HBD)	Polar Surface area (Å2)	No of rotatable bonds	Follows	Violation
VENETOCLAX	868.457	8.6599	−3.037	11	3	360.03	13	1	3
IRINOTECAN HYDROCHLORIDE	623.15	4.5129	−3.659	9	1	261.909	4	3	1
IMATINIB	493.615	4.59032	−3.021	7	2	216.956	7	4	0
ACALABRUTINIB	465.517	3.3126	−3.034	7	2	201.573	4	4	0
ZANUBRUTINIB	471.561	4.2226	−4.892	6	2	203.917	6	4	0
IBRUTINIB	440.507	4.2173	−3.501	7	1	190.873	5	4	0
SIROLIMUS	914.187	6.1806	−4.398	13	3	386.722	6	1	3
REGORAFENIB	482.821	5.6888	−4.324	4	3	189.277	5	3	1
BROMOCRIPTINE	654.606	3.1928	−3.462	6	3	259.451	5	3	1
PIMOZIDE	461.556	5.857	−2.899	3	1	197.397	7	3	1

Based on ADME and toxicity analyses, IMATINIB, IRINOTECAN HYDROCHLORIDE, and IBRUTINIB were identified as strong therapeutic candidates for pAML. Their Human Intestinal Absorption (HIA) scores, all above 90%, indicate excellent oral bioavailability and efficient absorption. Importantly, their Blood Brain Barrier (BBB) permeability remained below 0.3, suggesting minimal Central Nervous System (CNS) exposure and reduced neurotoxicity risk. Toxicity profiling further validated their safety. Collectively, these features—favorable absorption, restricted BBB passage, and low toxicity—highlight their therapeutic value. **Table 5** presents a detailed overview of each compound’s pharmacokinetic and safety properties.

**Table 5:** ADME/T profile of the top 10 drugs.

Compounds	Absorption			Distribution		Metabolism	Excretion	Toxicity		
	Caco2 Permeability	HIA (%)	P-gpI	BBB	CNS	CYP3A4 (Inhibitor)	TC	AMES	LC_50_ (log mM)	LD_50_ (mole/kg)
					(Permeability)					
VENETOCLAX	0.847	100	Yes	−1.747	−3.119	Yes	−0.096	No	−0.481	2.604
IRINOTECAN HYDROCHLORIDE	0.657	99.634	Yes	−1.439	−3.2	Yes	1.157	No	0.467	2.832
IMATINIB	1.092	93.847	Yes	−1.376	−2.514	Yes	0.716	No	2.089	2.9
ACALABRUTINIB	1.37	85.963	Yes	−1.154	−3.062	Yes	0.403	Yes	1.577	2.554
ZANUBRUTINIB	0.727	86.57	Yes	−0.814	−2.338	Yes	0.397	Yes	1.369	2.226
IBRUTINIB	0.704	97.724	Yes	−0.902	−2.431	Yes	0.601	No	−2.866	3.089
SIROLIMUS	0.567	62.002	Yes	−1.674	−2.941	No	0.558	No	4.078	0.285
REGORAFENIB	0.706	88.745	Yes	−1.676	−2.064	Yes	−0.042	No	−0.301	2.111
BROMOCRIPTINE	0.449	71.357	Yes	−0.711	−2.601	Yes	0.327	No	2.448	3.739
PIMOZIDE	0.121	84.897	Yes	0.004	0.487	Yes	0.631	Yes	2.339	2.442

#### 3.11.2. Cross-validation of candidate drugs.

We conducted molecular docking of our three suggested candidate drugs with 27 different receptors previously established as key genes (KGs) or hub genes (HGs) in published research to determine the binding affinities between these key receptors and our suggested compounds (S11 Table). Fig 6B presents the heatmap of these binding affinities.

#### 3.11.3. Validation of docking specificity using negative controls.

To validate the docking specificity, the binding affinity scores (BASs) of the original drug compounds were compared with the average BASs of the decoy molecules with each of the target receptors (S13 Table). In all cases, we observed that original BASs are more significant than the BASs with the decoy molecules. These differences supported the specificity of the docking results, indicating that the proposed drug-receptor interactions were not at random positions and there is no chance of selecting a decoy molecule as a drug molecule through BASs.

#### 3.11.4. Density functional theory (DFT) analysis.

Frontier molecular orbital (FMO) analysis was used to assess compound reactivity through HOMO-LUMO interactions (HOMO- Highest Occupied Molecular Orbital and LUMO- Lowest Unoccupied Molecular Orbital). A smaller HOMO-LUMO energy gap indicates greater reactivity and less chemical stability [71], influencing ligand-receptor binding dynamics.

IRINOTECAN HYDROCHLORIDE exhibits the lowest HOMO-LUMO energy gap (2.7122 eV) compared to IMATINIB (2.7754 eV) and IBRUTINIB (2.8447 eV), indicating higher chemical reactivity and potential for stronger molecular interactions. Besides, their moderate HOMO-LUMO gaps reflect ideal stability-reactivity profiles essential for their anticancer efficacy and therapeutic performance. DFT calculations further assessed the quantum chemical properties of these compounds (S14 Table). Among the three, IRINOTECAN HYDROCHLORIDE exhibits the highest electron affinity (5.5909 eV), suggesting a greater ability to accept electrons, which may enhance its reactivity and interaction with electron-rich biological targets compared to IMATINIB (5.239 eV) and IBRUTINIB (4.9794 eV). S5 Fig illustrates the visual representation of the HOMO-LUMO of these candidate drug molecules.

### 3.12 Molecular dynamics (MD) simulation

**Fig 7:**
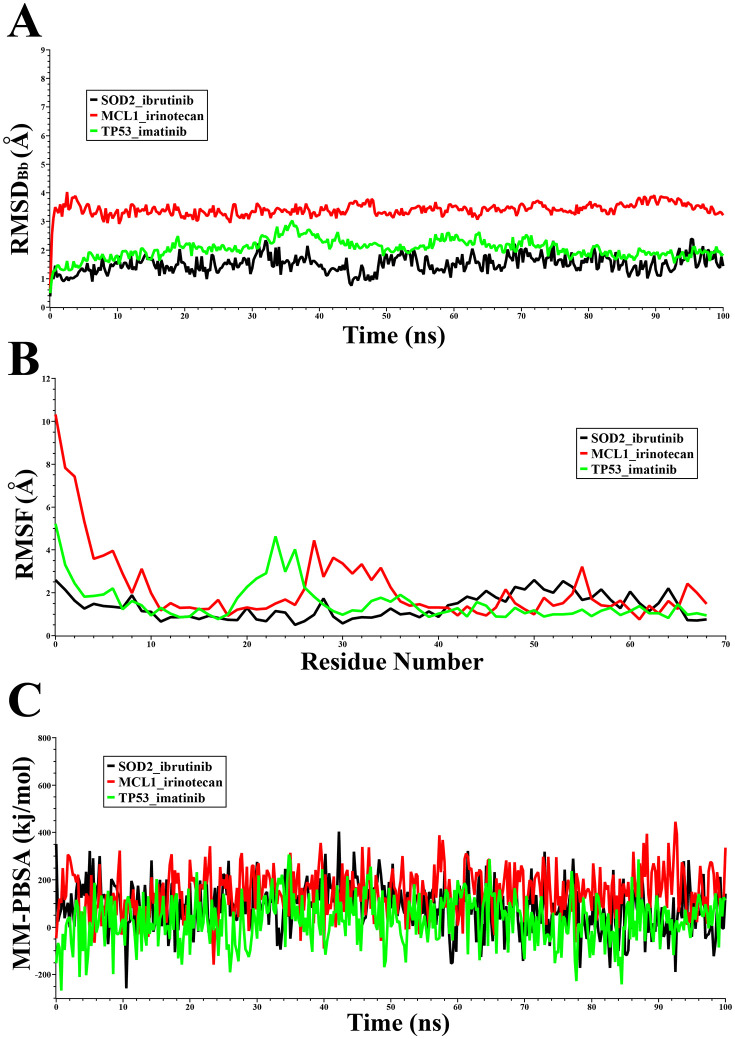
Molecular Dynamics (MD) simulation results: (A) the root means square deviation (RMSD), (B) the root mean square fluctuation (RMSF), and (C) the binding free energy (MM-PBSA) of the top-ranked drug-target complexes with three candidate drugs for a duration of 100-ns simulation.

Based on the analysis above, IBRUTINIB, IMATINIB and IRINOTECAN HYDROCHLORIDE met all drug-likeness and pharmacokinetic requirements, leading to their selection for 100 ns molecular dynamics (MD) simulations to assess stability. The evaluation focused on key metrics including RMSD, RMSF, and MM-PBSA binding free energy. RMSD indicates the average atomic displacement over time, reflecting complex stability; as depicted in Fig 5, all three drug-target complexes maintained stable configurations with average RMSD values of 1.51 Å for SOD2_ibrutinib, 3.41 Å for MCL1_irinotecan, and 2.04 Å for TP53_imatinib. Here, SOD2_ibrutinib exhibited the least fluctuation, suggesting greater stability (**Fig 7A**). RMSF analysis, which measures residue flexibility, showed SOD2_ibrutinib to be the most rigid complex with an average value of 1.35 Å, surpassing 2.19 Å for MCL1_irinotecan and 1.53 Å for TP53_imatinib, indicating more consistent target interactions (**Fig 7B**). MM-PBSA binding free energy, where higher positive ΔG denotes stronger binding in YASARA, revealed significant stability for all with average scores of 85.01 kJ/mol (SOD2_ibrutinib), 149.45 kJ/mol (MCL1_irinotecan), and 30.66 kJ/mol (TP53_imatinib). MCL1_irinotecan demonstrated the strongest binding, followed by SOD2_ibrutinib, with TP53_imatinib showing comparatively weaker interaction (**Fig 7C**). These findings confirm that the three drugs form stable, effective bindings with their receptors, supporting their potential as therapeutic agents against pAML.

## 4. Discussion

**Fig 8:**
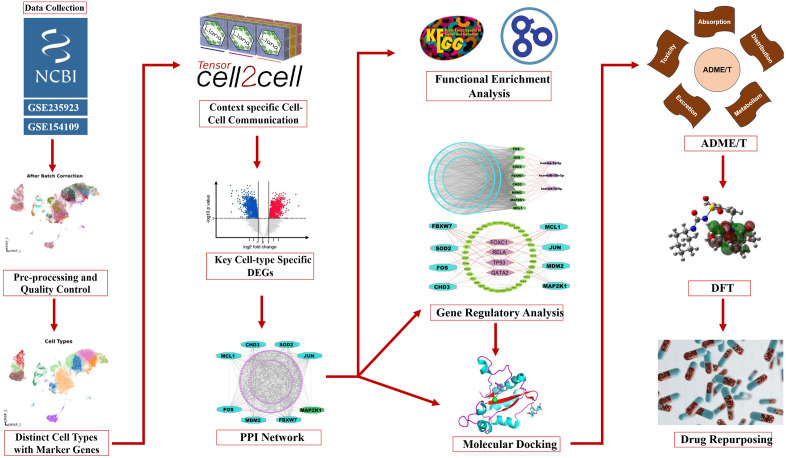
Graphical summary of this study.

Pediatric acute-myeloid-leukemia (pAML) is a highly aggressive hematologic malignancy that can be fatal without timely intervention [2]. For improved diagnosis and treatment strategies, this study analyzed scRNA-seq profiles using bioinformatics and system biology approaches. The graphical summary of this study was summarized in **Fig 8**.

At first, we performed quality control steps of scRNA-Seq profiles such as doublet removal, mitochondrial gene filtering, and other preprocessing. Then we found 55,503 high-quality cells from the raw count matrix. Then, a uniform cell distribution was ensured across samples through batch correction and visualized via UMAP. The Leiden algorithm identified 21 distinct cell clusters, each characterized by its top 20 marker genes ranked by highest log fold-change (logFC) values. Using these marker genes and curated databases, clusters were annotated into 12 cell types: Basophils, Dendritic cells, Erythroid cells, Gamma delta T cells (GdT), Neutrophils, Plasma cells, Progenitor cells, T regulatory cells (T-regs), Naive B cells, Naive CD4 T cells, Plasmacytoid dendritic cells (Plasmacytoid DCs), and Non-Classical Monocytes. We also validated the marker gene expression and visualized via matrix plot. Context-specific cell-cell communication (CCC) analysis between healthy and pAML samples identified six key cell types: Erythroid cells, GdT cells, Naive B cells, Naive CD4-T cells, Non-Classical Monocytes, and T-regs—showing differential ligand-receptor interactions. Then, to identify cell-type-specific cDEGs, we performed differential expression analysis of these key cell types. Consequently, eight common key genes (cKGs) were identified using a PPI network built via the STRING database, with seven (JUN, MDM2, FOS, SOD2, FBXW7, CHD3, MCL1) upregulated and one (MAP2K1) downregulated. The cKGs regulatory network analysis found FOXC1, GATA2, RELA, and TP53 as key transcriptional, and hsa-let-7a-5p, hsa-let-7e-5p, and hsa-miR-15a-5p as key post-transcriptional regulators of cKGs.

The *JUN* proto-oncogene encodes c-Jun, a key transcription factor with a basic leucine zipper domain, expressed in macrophages, neutrophils, and lymphocytes [72,73]. Upregulated *JUN*, triggered by MEK signaling, promotes pAML progression by activating the unfolded protein response (UPR) to help leukemic cells manage ER stress. It does so by binding to and enhancing transcription of key UPR genes like XBP1 and ATF4, supporting cell survival and preventing apoptosis [74]. Furthermore, c-JUN and c-FOS (encoded by *FOS* [75]) form the CC: transcription factor AP-1 complex, which is essential for cell proliferation and activation [76]. In AML, the upregulation of *SOD2* [77] and response to xenobiotic stimuli elevate reactive oxygen species (ROS) levels [78]. Though *SOD2* typically controls ROS by converting harmful superoxide radicals in mitochondrion, this balance is disrupted in pAML, leading to DNA damage, impaired blood cell development, and leukemic progression [79,80]. *MDM2* (murine double minute 2), a p53-specific E3 ubiquitin ligase, binds and degrades p53 via ubiquitination [81]. In AML, TP53 induces the transcription of *MDM2* [82] and GATA2 regulates the expression of RASSF4, a modulator of the p53 inhibitor *MDM2* [83]. The upregulation of *MDM2* lowers p53 levels, weakening tumor suppression and enabling leukemic cell survival and resistance to apoptosis [84]. Additionally, p53 regulates MAPK [85], a component of the endocrine resistance pathway, which promotes cell proliferation and inhibits apoptosis, supporting leukemic cell growth and survival [86]. Moreover, MAPK/ERK signaling regulates *MCL1* expression [87] which is upregulated in AML [88]. It promotes disease progression by supporting the self-renewal of leukemic stem cells and resistance to apoptosis [89,90]. Besides, Naa10p suppresses apoptosis by promoting *MCL1* transcription via its interaction with RELA [91]. Notably, it is a pivotal component of the nuclear factor kappa B (NF-κB) complex, which regulates various cellular processes, including survival, proliferation, and differentiation [92]. Similarly, other cKGs and their regulatory elements identified in this study play critical roles in activating signaling networks that drive pAML development [93–95]. Their combined activity contributes to the molecular mechanisms underlying disease progression. The identification of cKGs, and the underlying pathogenetic mechanisms of pAML enables drug repurposing as a targeted strategy to modulate dysregulated mechanisms and mitigate disease progression. The regulators hsa-let-7a-5p, hsa-let-7e-5p, and hsa-miR-15a-5p have important roles in leukemia at post-transcriptional level by controlling key pathways involved in cell proliferation, survival, and apoptosis [96,97]. The let-7 family inhibits the RAS/MAPK pathway, which lowers JUN and FOS activity. Low level of let-7 accumulate these proteins which promote leukemia cell growth by forming AP-1 [76]. Furthermore, it lowers the p53 activity and increase MDM2 leading to reduced apoptosis [84]. At the same time, miR-15a-5p suppresses MCL1 and controls MAPK1 [98,99]. miR -15a down-regulation allows an increase in the levels of MCL1 and MAP2K1, which protects leukemic cells against apoptosis and increases their survival [87,100].

FOS gene controls inflammatory responses triggered by IL-17. FOS gets involved in the IL-17 signaling pathway by participating in MAPK and AP-1 activation and thus contributing to immunity and inflammation regulation [101]. AP-1 transcription factor complex is essential for transcriptional processes triggered by RANKL that result in the development of osteoclasts and bone resorption. And together with JUN, FOS controls osteoclast differentiation by forming AP-1 transcription factor complex [102]. Moreover, MAP2K1, a kinase in the MAPK/ERK pathway, controls cellular senescence by phosphorylating cell cycle regulators p21 and p16 and thereby influencing cell cycle arrest and senescence-associated secretory phenotype (SASP) [103,104].

The molecular docking of 196 drug molecules with 12 target receptors, where eight are cKGs and four are TFs, helped us to identify three effective candidate drugs: IRINOTECAN HYDROCHLORIDE, IMATINIB, and IBRUTINIB for pAML treatment. These candidates demonstrated strong binding affinities, favorable drug-likeness according to Lipinski’s Rule, and excellent ADME/T profiles, including high oral bioavailability (HIA > 90%), minimal BBB permeability, and low predicted toxicity. Additionally, DFT analysis supported their molecular stability, revealing favorable HOMO-LUMO energy gaps (ΔE), chemical hardness (ɳ), and electrophilicity indices (ω), reinforcing their potential as effective therapeutic agents. We also evaluated these three drug candidates against 27 independent receptors reported as key or hub genes in published studies, which also exhibited strong binding, supporting our findings. Ethyl-10-hydroxy-camptothecin (SN38) is the active metabolite of IRINOTECAN HYDROCHLORIDE (CPT-11) [105]. It targets *MDM2* to activate TP53-mediated apoptosis and thereby effectively halts pAML progression [106]. IMATINIB, a tyrosine kinase inhibitor, targets c-KIT and disrupts downstream MAPK and AKT signaling pathways essential for leukemic cell survival and proliferation, making it a promising therapeutic candidate for leukemia [107]. IBRUTINIB effectively suppresses downstream NF-κB signaling pathways, which is crucial for leukemic cell survival and proliferation, and thus makes it a promising therapeutic candidate for pAML [108]. Current treatment of pediatric pAML relies predominantly on aggressive chemotherapy, and targeted therapy options are still limited. The suggested candidates represent a broader pAML therapeutic strategy away from sole cytotoxic chemotherapy to the targeted inhibition of kinase signaling and damage to pathways. IMATINIB is presently FDA approved and clinically proven in pediatric chronic myeloid leukemia (CML) and Philadelphia chromosome–positive acute lymphoblastic leukemia (Ph + ALL) [109]. In addition, IBRUTINIB has been very effective in chronic lymphocytic leukemia in adults, as indicated by phase III trials [110]. Interestingly, preclinical studies also detected activity of Ibrutinib in blasts from AML, which represents a promising biological rationale for further clinical evaluation of IBRUTINIB in AML [111]. Also, the combination of IRINOTECAN with high-dose cytarabine (HiDAC) demonstrated satisfactory response with acceptable toxicity in AML [112]. Therefore, their therapeutic value for pAML management requires further confirmation through laboratory studies and clinical research.

This research has important public health implications too. pAML relapse is a major issue worldwide, but particularly in low-resource settings where costly therapy is difficult to obtain [6,7]. By identifying signature genes and regulatory networks and highlighting affordable, repurposable drugs such as IMATINIB, IBRUTINIB, and IRINOTECAN HYDROCHLORIDE, our research contributes to development of superior targeted therapy that is accessible. Such strategies can decrease therapy failure, achieve higher survival rates in relapsed cases, and fill gaps in pAML therapy worldwide. Application of such findings in future clinical practice can lead to earlier therapy initiation and wider accessibility to most vulnerable children.

Though computational drug screening approaches (molecular docking, ADMET, DFT and MD simulation) reduce time, labor and cost in drug discovery, but they have certain limitations such approaches may not effective against off-target effects, disease heterogeneity, or drugs’ dynamic pharmacokinetics in the body that can lead to false positives or unappreciated toxicities. For this reason, experimental validation is crucial. The first step involves *in-vitro* testing of drug cytotoxicity on the target host cells in well-controlled experimental conditions [113]. Following this, *in-vivo* and ex-vivo analysis of animal models, e.g., Swiss albino mice, can evaluate the lead drug candidate’s safety and efficacy in an authentic biological setting [114]. Moreover, gene expression using quantitative PCR can confirm how such drugs influence the target proteins identified in this research [115].

## 5. Conclusion

This study identified pAML-causing common key genes (cKGs) across key cell types highlighting pathogenetic processes and therapeutic candidates through integrated scRNA-seq profile analysis. We identified twelve cell types. Among these, six cell types stood out as key types through context-specific cell-cell communication: Erythroid cells, GdT cells, Naive B cells, Naive CD4 T cells, Non-Classical Monocytes, and T-regs. From these key cell populations, we identified eight common key genes cKGs (JUN, MDM2, FOS, SOD2, FBXW7, CHD3, MCL1, and MAP2K1), which play essential roles in biological processes, signaling pathways, and regulatory networks associated with pAML. Disease-cKGs enrichment analysis demonstrated that these cKGs are not only linked to pAML but also showed significant associations with other leukemic diseases. The functional enrichment analysis further connected these genes to critical Gene Ontology (GO) terms and KEGG pathways relevant to pAML pathogenesis. Additionally, regulatory network analysis uncovered four key transcription factors (FOXC1, GATA2, RELA, and TP53) and three microRNAs (hsa-let-7a-5p, hsa-let-7e-5p, and hsa-miR-15a-5p) that modulate the expression of these cKGs. Using these genes as therapeutic targets, we computationally screened drugs and identified three high-potential candidates: IRINOTECAN HYDROCHLORIDE, IMATINIB, and IBRUTINIB. These were rigorously evaluated through molecular docking, ADMET profiling, and density functional theory (DFT) analysis, with support from existing literature. Thus, the findings provide important insights into pAML pathogenesis and potential therapeutic candidates.

## Supporting information

S1 File Data processing and integration.
(DOCX)

S2 File Cell clustering. (DOCX)

S3 File Identification of cluster-specific marker genes and corresponding cell types. (DOCX)

S4 File Identification of cell type specific common DEGs (cDEGs). (DOCX)

S5 File Molecular docking. (DOCX)

S6 File Density functional theory (DFT). (DOCX)

S7 File Molecular dynamics (MD) simulation. (DOCX)

S1 Table 31 Samples with population characteristics and total gene counts across all cells before and after quality control. (DOCX)

S2 Table Log fold change values across 21 communities.
(DOCX)

S3 Table Cell type specificity scores and logFC values for evaluating top 20 marker genes’ exclusivity in identifying specific cell types. (DOCX)

S4 Table Context-specific cell-cell communication matrix between pAML and healthy samples. (DOCX)

S5 Table List of upregulated and downregulated common DEGs (cDEGs) across key cell types. (DOCX)

S6 Table List of common key genes (cKGs) from the PPI network based on different topological measures. (DOCX)

S7 Table Association of cKGs with different diseases. (DOCX)

S8 Table Collection of pAML-related candidate drugs agents. (DOCX)

S9 Table Docking scores (binding affinities, kcal/mol) between the proposed receptors and top-ranked 30 candidate drugs (out of 196). (DOCX)

S10 Table Receptors identified as key genes (KGs) or hub genes (HGs) in the published literatures. (DOCX)

S11 Table Docking scores (binding affinities, kcal/mol) between the proposed drug candidates and 27 different receptors identified as key genes (KGs) or hub genes (HGs) in the published literatures. (DOCX)

S12 Table The identifiers of the Decoy molecules generated for IMATINIB, IBRUTINIB, and IRINOTECAN HYDROCLORIDE. (DOCX)

S13 Table The average binding affinity scores (BASs) of the decoy molecules and the BAS of suggested drug candidates in kcal/mol with the target receptors. (DOCX)

S14 Table Determined FMO energies along with their corresponding physicochemical descriptors, such as chemical hardness, electronegativity, softness, chemical potential, and global electrophilicity index. (DOCX)

S1 Fig UMAP plot of scRNA-seq data. (A) Sample-specific clustering observed before batch correction; (B) Uniform cell distribution achieved after batch correction. Here, each dot represents a cell, and each color represents a distinct sample.
(DOCX)

S2 Fig UMAP plot of cell types clustering for Harmony integration. (DOCX)

S3 Fig Matrix plot displaying marker genes of corresponding cell types. Rows and top column annotations represent cell types, while the lower columns correspond to their associated marker genes. Color intensity indicates column-scaled expression levels, with darker shades reflecting higher expression.
(DOCX)

S4 Fig Venn diagram of cDEGs among six key cell types. (A) Venn diagram of upregulated cDEGs among six key cell types that shows 154 upregulated cDEGs and (B) Venn diagram of downregulated cDEGs among six key cell types that shows 44 downregulated cDEGs.
(DOCX)

S5 Fig The HOMO and LUMO orbitals of the proposed drug molecules. (DOCX)
