# Supplementary material for: In-Silico discovery of Pediatric Acute-Myeloid-Leukemia (pAML) causing druggable molecular signatures highlighting their pathogenetic processes and therapeutic agents through single-cell RNA-Seq profile analysis
Source: PLoS One. 2025 Oct 31;20(10):e0335410. doi: 10.1371/journal.pone.0335410 (PMC12578151; doi:10.1371/journal.pone.0335410)
Supplement: S1 File — Data processing and integration. (DOCX) [file pone.0335410.s001.docx]

S1 Method. Data processing and integration

The Scanpy, a Python based toolkit was used to process the scRNA-seq data in our study. It is a comprehensive tools for preprocessing, visualization, clustering, and differential expression analysis [1]. We utilized Scanpy (version 1.11.0) in Python 3.11.5. After processing the data and doublet (instances where two cells may have been incorrectly captured together) removal, we filtered out the cells with fewer than 200 detected genes, genes expressed in fewer than three cells, and cells with over 10% mitochondrial gene expression to eliminate low-quality or damaged cells. MALAT1 was removed to prevent clustering bias. To ensure data quality, 99% of total counts were retained, and 31 samples were merged, keeping only consistently expressed genes. We normalized cells to a total count of 1×10⁴ using ‘scanpy.pp.normalize_total’ and performed log transformation via "scanpy.pp.log1p" to stabilize variance in gene expression values. To integrate the data scVI (version 1.0) was utilized [2]. We checked the robustness of our integration strategy by compareing scVI to the alternative tool Harmony. Also, we evaluated their performance in quantitative terms, such as Normalized Mutual Information (NMI) and graph connectivity. NMI measures clustering overlap, while the graph connectivity metric evaluates whether integrated data links all cells of the same identity [3]. To calculate NMI, we used ‘normalized_mutual_information_info_score’ and for graph connectivity score, we incorporated ‘scib.metics.graph_connectivity’ function. We then identified 5000 highly variable genes and retrieved approximately 55,503 high-quality single-cell transcriptomes for downstream analysis. Finally, highly variable genes were identified using ‘scanpy.pp.highly_variable_genes’.

**References**

1. Wolf FA, Angerer P, Theis FJ. SCANPY: large-scale single-cell gene expression data analysis. Genome Biol 2018; 19:1–5

2. Lopez R, Regier J, Cole MB, et al. Deep generative modeling for single-cell transcriptomics. Nat Methods 2018; 15:1053–1058

3. Luecken MD, Büttner M, Chaichoompu K, et al. Benchmarking atlas-level data integration in single-cell genomics. Nat Methods 2022; 19:41–50
