# Supplementary material for: In-Silico discovery of Pediatric Acute-Myeloid-Leukemia (pAML) causing druggable molecular signatures highlighting their pathogenetic processes and therapeutic agents through single-cell RNA-Seq profile analysis
Source: PLoS One. 2025 Oct 31;20(10):e0335410. doi: 10.1371/journal.pone.0335410 (PMC12578151; doi:10.1371/journal.pone.0335410)
Supplement: S2 File — (DOCX) [file pone.0335410.s002.docx]

S2 Method. Cell clustering

The neighborhood graph was constructed using default parameters in ‘scanpy.pp.neighbor’ function to map relationships. Uniform Manifold Approximation and Projection (UMAP) used to visualize the high-dimensional neighborhood graph in 2D, is a manifold learning technique to reduce dimensionality [1]. It is able to preserve more of the global structure with superior run time performance [2]. Furthermore, for Leiden algorithm [3], we incorporated the function ‘scanpy.tl.leiden’ function with a resolution parameter of 0.8 [4]. The marker genes were extracted via ‘scanpy.get.rank_genes_groups_df’ function in the differential expression analysis.

**References**

1. Becht E, McInnes L, Healy J, et al. Dimensionality reduction for visualizing single-cell data using UMAP. Nat Biotechnol 2019; 37:38–44

2. McInnes L, Healy J, Melville J. Umap: Uniform manifold approximation and projection for dimension reduction. arXiv preprint arXiv:1802.03426 2018;

3. Sahu S, Kothapalli K, Banerjee DS. Fast Leiden Algorithm for Community Detection in Shared Memory Setting. Proceedings of the 53rd International Conference on Parallel Processing 2024; 11–20

4. . From Louvain to Leiden: guaranteeing well-connected communities. Sci Rep 2019; 9:1–12
