# Supplementary material for: In-Silico discovery of Pediatric Acute-Myeloid-Leukemia (pAML) causing druggable molecular signatures highlighting their pathogenetic processes and therapeutic agents through single-cell RNA-Seq profile analysis
Source: PLoS One. 2025 Oct 31;20(10):e0335410. doi: 10.1371/journal.pone.0335410 (PMC12578151; doi:10.1371/journal.pone.0335410)
Supplement: S3 File — (DOCX) [file pone.0335410.s003.docx]

S3 Method. Identification of cluster-specific marker genes and corresponding cell types

The differential expression analysis was carried out for the cell-type-specific markers. We implemented ‘sc.get.rank_genes_gorups_df’ incorporating Wilcoxon test. It retrieves ranked gene list and associated statistics for each cell. The Wilcoxon rank-sum test is a non-parametric test that does not assume normal distribution of gene expression values. scRNA-seq data are inherently sparse and zero-inflated, making non-parametric approaches more appropriate for detecting robust differences in expression across cell clusters [1]. Therefore, we used it in our analysis. Genes with *p*-value < 0.05 and |logFC| > 1 were considered differentially expressed genes (DEGs). To further assess the specificity of the marker genes in specific cell. The cell-type specificity helps us to quantify and understand uniqueness of the marker genes to particular cell population.

**References**

1. Das S, Rai A, Merchant ML, et al. A comprehensive survey of statistical approaches for differential expression analysis in single-cell RNA sequencing studies. Genes (Basel) 2021; 12:1947
