# Supplementary material for: In-Silico discovery of Pediatric Acute-Myeloid-Leukemia (pAML) causing druggable molecular signatures highlighting their pathogenetic processes and therapeutic agents through single-cell RNA-Seq profile analysis
Source: PLoS One. 2025 Oct 31;20(10):e0335410. doi: 10.1371/journal.pone.0335410 (PMC12578151; doi:10.1371/journal.pone.0335410)
Supplement: S4 File — (DOCX) [file pone.0335410.s004.docx]

S4 Method. Identification of cell type specific common DEGs (cDEGs)

To find out the cDEGs across the key cell types, we performed differential expression analysis for all annotated cells. We carried out the test using t-test. The t-test assumes approximate normality after log-transformation and provides a sensitive method to detect mean differences in expression between groups. It is commonly used in scRNA-seq DEG workflows to complement non-parametric tests and provide reproducible results when applied to large sample sizes. In our study, the t-test was performed using ‘de.test.t_test’ function in Python. The function belongs to the diffxpy package. Significant differentially expressed genes (DEGs) were filtered using thresholds of *p*-value < 0.05 and |logFC| > 1. After getting the DEGs for the key cell types, we extracted the common DEGs (cDEGs) which were present in all key cell types and visualized using Jvenn [1].

**References**

1. Bardou P, Mariette J, Escudié F, et al. jvenn: an interactive Venn diagram viewer. BMC Bioinformatics 2014; 15:1–7
