# Supplementary material for: In-Silico discovery of Pediatric Acute-Myeloid-Leukemia (pAML) causing druggable molecular signatures highlighting their pathogenetic processes and therapeutic agents through single-cell RNA-Seq profile analysis
Source: PLoS One. 2025 Oct 31;20(10):e0335410. doi: 10.1371/journal.pone.0335410 (PMC12578151; doi:10.1371/journal.pone.0335410)
Supplement: S5 File — (DOCX) [file pone.0335410.s005.docx]

S5 Method. Molecular docking

Molecular docking is a widely applied computational technique used to screen large libraries of compounds for their potential to inhibit disease-associated genes. In this study, we utilized molecular docking to identify therapeutic molecules guided by common key genes (cKGs) relevant to pAML [1,2]. Specifically, our docking analyses targeted receptor proteins influenced by the cKGs as well as their associated regulatory transcription factors (TFs). After performing molecular docking, we ranked the receptors and ligands as following to get the top ranked candidate drug:
The molecular docking provided the binding affinity scores (BAS) between the target receptors and drug molecules. Let, B_ij_ be the BAS between the i^th^ receptor (i=1, 2, …, m) and j^th^ drug (j=1, 2, …, n). To prioritize receptors and drugs, we calculated the average binding affinity scores across each receptor and each drug. Then, we ranked the receptors in descending order based on their average BAS $\left( \frac{1}{m}\sum_{j=1}^{n} B_{ij}, i=1,2\ldots,m \right)$ and same for the drugs $\left( \frac{1}{n}\sum_{i=1}^{m} B_{ij}, j=1,2\ldots,n \right)$.

**References**

1. Reza MS, Hossen MA, Harun-Or-Roshid M, et al. Metadata analysis to explore hub of the hub-genes highlighting their functions, pathways and regulators for cervical cancer diagnosis and therapies. Discover Oncology 2022; 13:79

2. Kabir SR, Islam T, Mollah MNH. 2, 4-Dipropylphloroglucinol inhibits the growth of human lung and colorectal cancer cells through induction of apoptosis. Medical Oncology 2023; 40:129
