# Supplementary material for: In-Silico discovery of Pediatric Acute-Myeloid-Leukemia (pAML) causing druggable molecular signatures highlighting their pathogenetic processes and therapeutic agents through single-cell RNA-Seq profile analysis
Source: PLoS One. 2025 Oct 31;20(10):e0335410. doi: 10.1371/journal.pone.0335410 (PMC12578151; doi:10.1371/journal.pone.0335410)
Supplement: S6 File — (DOCX) [file pone.0335410.s006.docx]

S6 Method. Density functional theory (DFT)

The mathematical definitions of the required parameters are [1]:

HOMO energy = E_HOMO_

LUMO energy = E_LUMO_

Energy gap, ΔE = E_LUMO_ - E_HOMO_

Ionization potential (I) = - E_HOMO_

Electron affinity (A) = - E_LUMO_

Chemical hardness (η) = $\frac{I-A}{2}$

Softness (σ) = $\frac{1}{\eta}$

Electro-negativity (χ) = $\frac{I+A}{2}$

Chemical potential (µ) = - χ

**References**

1. El-Shamy NT, Alkaoud AM, Hussein RK, et al. DFT, ADMET and Molecular Docking Investigations for the Antimicrobial Activity of 6, 6′-Diamino-1, 1′, 3, 3′-tetramethyl-5, 5′-(4-chlorobenzylidene) bis [pyrimidine-2, 4 (1H, 3H)-dione]. Molecules 2022; 27:620
