# Supplementary material for: In-Silico discovery of Pediatric Acute-Myeloid-Leukemia (pAML) causing druggable molecular signatures highlighting their pathogenetic processes and therapeutic agents through single-cell RNA-Seq profile analysis
Source: PLoS One. 2025 Oct 31;20(10):e0335410. doi: 10.1371/journal.pone.0335410 (PMC12578151; doi:10.1371/journal.pone.0335410)
Supplement: S7 File — (DOCX) [file pone.0335410.s007.docx]

S7 Method. Molecular dynamics (MD) simulation

Molecular Dynamics (MD) simulations were performed to support and validate the findings from molecular docking. MD simulations provide critical insights into the stability of protein-ligand complexes by capturing their dynamic behavior, including fluctuations and conformational changes, as the system equilibrates over time [1]. We conducted Molecular dynamics (MD) simulations of the top protein–ligand complexes using YASARA software over a timeframe of 100-ns with the AMBER14 force field to assess their dynamic behavior. Three complexes with the best docking scores were selected and simulated for 100ns under certain physiological conditions. Each complex was solvated with a TIP3P water model [2], and hydrogen bonds were optimized. Periodic boundary conditions maintained solvent density at 0.997 g/L. Energy minimization was done using simulated annealing and steepest descent over 5,000 cycles. Simulations were run for 100 ns under physiological conditions (298 K, pH 7.4, 0.9% NaCl) using a 2.5 fs [3] time-step and the Berendsen thermostat [4]. After the simulation, trajectories were saved every 100 ps for analysis with YASARA macros [5] and SciDAVis (<http://scidavis.sourceforge.net/>). Key metrics including Root Mean Square Deviation (RMSD), Residue Root Mean Square Fluctuation (RMSF), and MM-PBSA binding free energy (ΔG_bind_) were calculated to evaluate the stability and dynamic behavior of the protein-ligand complexes throughout the molecular dynamics simulations. The ΔG_bind_ was calculated as: ΔG_bind_ = E_complex_ – (E_protein_ + E_ligand_).

**References**

1. Kaya G, Noma SAA, Barut Celepci D, et al. Design, synthesis, spectroscopic characterizations, single crystal X-ray analysis, in vitro xanthine oxidase and acetylcholinesterase inhibitory evaluation as well as in silico evaluation of selenium-based N-heterocyclic carbene compounds. J Biomol Struct Dyn 2023; 41:11728–11747

2. Jorgensen WL, Chandrasekhar J, Madura JD, et al. Comparison of simple potential functions for simulating liquid water. J Chem Phys 1983; 79:926–935

3. Krieger E, Nielsen JE, Spronk CAEM, et al. Fast empirical pKa prediction by Ewald summation. J Mol Graph Model 2006; 25:481–486

4. Berendsen HJC, Postma JPM van, Van Gunsteren WF, et al. Molecular dynamics with coupling to an external bath. J Chem Phys 1984; 81:3684–3690

5. Krieger E, Koraimann G, Vriend G. Increasing the precision of comparative models with YASARA NOVA—a self‐parameterizing force field. Proteins: Structure, Function, and Bioinformatics 2002; 47:393–402
