# Supplementary material for: In-Silico discovery of Pediatric Acute-Myeloid-Leukemia (pAML) causing druggable molecular signatures highlighting their pathogenetic processes and therapeutic agents through single-cell RNA-Seq profile analysis
Source: PLoS One. 2025 Oct 31;20(10):e0335410. doi: 10.1371/journal.pone.0335410 (PMC12578151; doi:10.1371/journal.pone.0335410)
Supplement: S1 Table — (DOCX) [file pone.0335410.s008.docx]

## **S1 Table.** 31 Samples with population characteristics and total gene counts across all cells before and after quality control.

| **Accession No.** | **Sample** | **Disease state** | **Number of cells before preprocessing** | **Number of genes before preprocessing** | **Number of cells after preprocessing** | **Number of genes after preprocessing** |
| --- | --- | --- | --- | --- | --- | --- |
| **GSE154109** | GSM4664009 | Healthy | 1687 | 33538 | 1410 | 14724 |
|  | GSM4664010 | Healthy | 1022 | 33538 | 875 | 13482 |
|  | GSM4664011 | Healthy | 1586 | 33538 | 1370 | 14192 |
|  | GSM4664012 | Healthy | 1687 | 33538 | 1451 | 14226 |
|  | GSM4664013 | pAML | 1472 | 33538 | 1133 | 15536 |
|  | GSM4664014 | pAML | 518 | 33538 | 182 | 12007 |
|  | GSM4664015 | pAML | 1173 | 33538 | 818 | 14382 |
|  | GSM4664016 | pAML | 1947 | 33538 | 1393 | 13894 |
|  | GSM4664017 | pAML | 2137 | 33538 | 1363 | 15742 |
|  | GSM4664018 | pAML | 1669 | 33538 | 289 | 13757 |
|  | GSM4664019 | pAML | 1171 | 33538 | 930 | 15555 |
|  | GSM4664020 | pAML | 2341 | 33538 | 1779 | 15033 |
| **GSE235923** | GSM7511998 | pAML | 4265 | 33538 | 3735 | 15591 |
|  | GSM7511999 | pAML | 3495 | 33538 | 2583 | 16057 |
|  | GSM7512000 | pAML | 2679 | 33538 | 1295 | 16381 |
|  | GSM7512002 | pAML | 4268 | 33538 | 3341 | 17013 |
|  | GSM7512003 | pAML | 2964 | 33538 | 545 | 15149 |
|  | GSM7512006 | pAML | 9588 | 33538 | 2048 | 16716 |
|  | GSM7512009 | pAML | 3457 | 33538 | 2886 | 15217 |
|  | GSM7512010 | pAML | 3057 | 33538 | 1138 | 13263 |
|  | GSM7512011 | pAML | 3965 | 33538 | 2683 | 15045 |
|  | GSM7512012 | pAML | 1431 | 33538 | 530 | 13080 |
|  | GSM7512013 | pAML | 3047 | 33538 | 1277 | 14773 |
|  | GSM7512014 | pAML | 2276 | 33538 | 480 | 14369 |
|  | GSM7512015 | pAML | 3821 | 33538 | 3033 | 14815 |
|  | GSM7512016 | pAML | 4134 | 33538 | 2925 | 15925 |
|  | GSM7512019 | pAML | 4278 | 36601 | 2872 | 19545 |
|  | GSM7512021 | pAML | 3442 | 36601 | 2492 | 18537 |
|  | GSM7512023 | pAML | 4848 | 36601 | 3349 | 18457 |
|  | GSM7512025 | pAML | 4430 | 36601 | 3048 | 18602 |
|  | GSM7512027 | pAML | 5041 | 36601 | 3259 | 18343 |
