# Supplementary material for: In-Silico discovery of Pediatric Acute-Myeloid-Leukemia (pAML) causing druggable molecular signatures highlighting their pathogenetic processes and therapeutic agents through single-cell RNA-Seq profile analysis
Source: PLoS One. 2025 Oct 31;20(10):e0335410. doi: 10.1371/journal.pone.0335410 (PMC12578151; doi:10.1371/journal.pone.0335410)
Supplement: S2 Table — Log fold change values across 21 communities. (DOCX) [file pone.0335410.s009.docx]

## **S2 Table.** Log fold change values across 21 communities.

| **C0**  **community** | **C0**  **logFC** |  | **C1**  **community** | **C1**  **logFC** |  | **C2**  **community** |  | **C2**  **logFC** |  | **C3**  **community** | **C3**  **logFC** |
| --- | --- | --- | --- | --- | --- | --- | --- | --- | --- | --- | --- |
| ADAMTS10 | 3.6725 |  | RECQL4 | 3.9481 |  | LYZ |  | 5.5290 |  | MPO | 4.0667 |
| SOCS2 | 3.4574 |  | MYBL2 | 3.3921 |  | AC020656.1 |  | 4.4015 |  | PRSS57 | 3.7675 |
| GOLGA8N | 3.3938 |  | SAPCD2 | 3.2217 |  | SMARCD3 |  | 3.9043 |  | EGFL7 | 3.2743 |
| AGRN | 3.2876 |  | CENPM | 3.2161 |  | S100A11 |  | 3.7539 |  | NPW | 3.2499 |
| NDRG2 | 3.1344 |  | CDKN2A | 3.2126 |  | CST3 |  | 3.7420 |  | CDCA7 | 3.2125 |
| OSM | 3.0191 |  | FOXM1 | 3.2000 |  | S100A6 |  | 3.5637 |  | TM7SF3 | 2.7284 |
| PLD4 | 2.9949 |  | WDR90 | 3.1373 |  | FCER1G |  | 3.5315 |  | FAM216A | 2.6669 |
| COL9A2 | 2.9554 |  | WDR62 | 3.0436 |  | PTAFR |  | 3.5088 |  | MGLL | 2.6522 |
| AZU1 | 2.9331 |  | AZU1 | 3.0144 |  | TYROBP |  | 3.4623 |  | STMN1 | 2.6288 |
| PRMT7 | 2.8871 |  | E2F1 | 2.9850 |  | NCF1 |  | 3.3086 |  | MGST1 | 2.5664 |
| AP1S3 | 2.7482 |  | CHTF18 | 2.9598 |  | LILRB3 |  | 3.2912 |  | FAM30A | 2.5244 |
| LCAT | 2.7204 |  | CEP55 | 2.9546 |  | MNDA |  | 3.2756 |  | AC002454.1 | 2.5179 |
| RFLNB | 2.7021 |  | BIRC5 | 2.9381 |  | ITGAM |  | 3.2669 |  | AMN | 2.5151 |
| SLC15A2 | 2.6855 |  | SHCBP1 | 2.9311 |  | CKAP4 |  | 3.2346 |  | CD38 | 2.4819 |
| KIAA0930 | 2.6717 |  | CDCA5 | 2.9174 |  | TYMP |  | 3.2345 |  | NUCB2 | 2.4509 |
| GRK2 | 2.6498 |  | RNASE3 | 2.9037 |  | S100A4 |  | 3.2294 |  | SYNGR1 | 2.4406 |
| KBTBD11 | 2.5751 |  | PKMYT1 | 2.8958 |  | CTSS |  | 3.2071 |  | VSIG10 | 2.4053 |
| LRFN4 | 2.4398 |  | RAD54L | 2.8617 |  | CLEC7A |  | 3.1851 |  | MZB1 | 2.4052 |
| ABCA2 | 2.4197 |  | TIMELESS | 2.7990 |  | LINC00937 |  | 3.1317 |  | SLC2A5 | 2.4007 |
| CLIP2 | 2.4059 |  | POLQ | 2.7954 |  | CSTA |  | 3.1301 |  | CASP6 | 2.3972 |

| **C4**  **community** | **C4**  **logFC** |  | **C5**  **community** | **C5**  **logFC** |  | **C6**  **community** | **C6**  **logFC** |  | **C7**  **community** | **C7**  **logFC** |
| --- | --- | --- | --- | --- | --- | --- | --- | --- | --- | --- |
| CRIP2 | 3.2528 |  | LINC02446 | 6.1909 |  | CST3 | 3.5354 |  | GZMH | 9.4711 |
| EGFL7 | 2.9098 |  | LEF1 | 5.6577 |  | CEBPD | 3.4618 |  | FGFBP2 | 8.9508 |
| MTRNR2L8 | 2.8467 |  | TRABD2A | 5.2297 |  | TREM1 | 3.3432 |  | GZMA | 8.5947 |
| MPO | 2.8369 |  | NELL2 | 5.0898 |  | EMP1 | 3.2006 |  | KLRD1 | 8.3645 |
| CD99 | 2.8070 |  | CCR7 | 5.0333 |  | GPAT3 | 3.1598 |  | GZMB | 7.9803 |
| AREG | 2.7332 |  | CAMK4 | 4.9601 |  | HMOX1 | 3.1139 |  | GNLY | 7.7578 |
| TSC22D1 | 2.6648 |  | MAL | 4.8143 |  | CLEC7A | 2.9712 |  | PRF1 | 7.6153 |
| PDLIM1 | 2.5665 |  | CD3E | 4.7714 |  | PHLDA1 | 2.9330 |  | CCL5 | 6.5807 |
| FHL1 | 2.4668 |  | CD8B | 4.6983 |  | AREG | 2.9330 |  | KLRB1 | 6.5626 |
| LUZP1 | 2.4550 |  | IL7R | 4.4816 |  | UPP1 | 2.9099 |  | GZMK | 6.5192 |
| PRSS57 | 2.4455 |  | BCL11B | 4.3777 |  | TBC1D8 | 2.8808 |  | IL2RB | 5.8837 |
| JUP | 2.3475 |  | CD3D | 4.2843 |  | IER3 | 2.7873 |  | NKG7 | 5.5522 |
| SMIM3 | 2.3362 |  | TCF7 | 4.2086 |  | SGK1 | 2.6890 |  | CST7 | 5.5359 |
| SERPINB1 | 2.2954 |  | CD3G | 4.1363 |  | MIR22HG | 2.6852 |  | CCL4 | 5.5098 |
| FAM30A | 2.2761 |  | LTB | 4.1152 |  | LYZ | 2.6257 |  | SAMD3 | 5.2251 |
| TPPP3 | 2.2746 |  | TRAC | 4.0989 |  | SPHK1 | 2.6165 |  | GZMM | 5.0978 |
| SPACA9 | 2.2704 |  | CD27 | 3.9620 |  | SAT1 | 2.5813 |  | KLRG1 | 4.8505 |
| THBS3 | 2.1940 |  | OXNAD1 | 3.8758 |  | GPR183 | 2.5738 |  | MYOM2 | 4.8195 |
| FSCN1 | 2.1825 |  | ITK | 3.8623 |  | LINC01678 | 2.5413 |  | NCR3 | 4.7619 |
| NPDC1 | 2.1797 |  | APBA2 | 3.8547 |  | PIK3R5 | 2.5072 |  | IL32 | 4.6708 |

| **C8**  **community** | **C8**  **logFC** |  | **C9**  **community** | **C9**  **logFC** |  | **C10**  **community** | **C10**  **logFC** |  | **C11**  **community** | **C11**  **logFC** |
| --- | --- | --- | --- | --- | --- | --- | --- | --- | --- | --- |
| FCGR3A | 6.6160 |  | MS4A1 | 8.2593 |  | MPO | 4.3392 |  | CSF1 | 4.3898 |
| CDKN1C | 5.2161 |  | CD79A | 7.0533 |  | ABHD4 | 3.7548 |  | SLC45A3 | 4.2050 |
| LILRA1 | 4.6448 |  | TNFRSF13C | 6.7686 |  | NPW | 3.4905 |  | MTRNR2L12 | 3.5001 |
| SIGLEC10 | 4.6326 |  | CD22 | 6.4351 |  | UBE2C | 2.9263 |  | MS4A3 | 3.3326 |
| RRAS | 4.5604 |  | IGHD | 6.3198 |  | UHRF1 | 2.8647 |  | REXO5 | 3.1000 |
| LILRB1 | 4.5306 |  | IGHM | 6.1617 |  | PRSS57 | 2.7932 |  | RAB33A | 3.0289 |
| LILRB2 | 4.4763 |  | BANK1 | 5.4556 |  | CDK1 | 2.7799 |  | TESPA1 | 2.9664 |
| FCER1G | 4.4066 |  | LINC00926 | 5.1688 |  | STMN1 | 2.7476 |  | HACD1 | 2.9057 |
| LST1 | 4.3266 |  | FCER2 | 4.8298 |  | UNG | 2.7456 |  | SOX4 | 2.7564 |
| S100A11 | 4.2668 |  | PNOC | 4.7830 |  | CDC20 | 2.6695 |  | FTO | 2.7241 |
| HMOX1 | 4.2267 |  | RALGPS2 | 4.6796 |  | FARSA | 2.6477 |  | ANKRD28 | 2.7203 |
| CTSS | 4.1563 |  | IGKC | 4.4830 |  | KIF20A | 2.6264 |  | CTNNBL1 | 2.6509 |
| AIF1 | 3.9884 |  | SPIB | 4.3857 |  | DEPDC1B | 2.6186 |  | NLK | 2.6482 |
| BCL2A1 | 3.9362 |  | RAB30 | 4.2330 |  | TYMS | 2.6033 |  | STXBP5 | 2.6363 |
| PILRA | 3.8918 |  | CD40 | 4.2195 |  | RAD54L | 2.5927 |  | GDF11 | 2.5337 |
| MARCKS | 3.8882 |  | CD79B | 4.1050 |  | MS4A3 | 2.5688 |  | TSC22D1 | 2.4988 |
| CEBPB | 3.8773 |  | SNX22 | 3.9869 |  | COCH | 2.5207 |  | HIST2H2BE | 2.4388 |
| C3AR1 | 3.6850 |  | P2RX5 | 3.9655 |  | MGST1 | 2.5198 |  | AC012306.2 | 2.4380 |
| IFITM3 | 3.6295 |  | CCR6 | 3.8418 |  | GINS1 | 2.4386 |  | DST | 2.4166 |
| MS4A7 | 3.5548 |  | HVCN1 | 3.8099 |  | OIP5 | 2.4182 |  | MIPEP | 2.3785 |

| **C12**  **community** | **C12**  **logFC** |  | **C13**  **community** | **C13**  **logFC** |  | **C14**  **community** | **C14**  **logFC** |  | **C15**  **community** | **C15**  **logFC** |
| --- | --- | --- | --- | --- | --- | --- | --- | --- | --- | --- |
| IL32 | 4.7904 |  | SNCA | 8.3379 |  | CA2 | 6.9674 |  | TPM2 | 5.4239 |
| LTB | 4.4072 |  | SLC25A37 | 8.1769 |  | PRDX2 | 6.4672 |  | DERL3 | 5.0008 |
| CD3E | 4.3533 |  | TRIM58 | 7.9442 |  | DLGAP5 | 6.2679 |  | SPIB | 4.8896 |
| IL7R | 4.3339 |  | BPGM | 7.9348 |  | BLVRB | 5.9519 |  | C12orf75 | 4.8545 |
| GATA3 | 4.0153 |  | DCAF12 | 7.9027 |  | TUBG1 | 5.8527 |  | IRF4 | 4.6750 |
| KLRB1 | 3.9316 |  | FAM210B | 7.4871 |  | HMBS | 5.6615 |  | AL096865.1 | 4.5373 |
| CD5 | 3.8798 |  | BLVRB | 7.3745 |  | NUSAP1 | 5.4931 |  | MZB1 | 4.2199 |
| CD3D | 3.8681 |  | FECH | 7.1273 |  | KIF4A | 5.3857 |  | UGCG | 4.2140 |
| CCR6 | 3.8612 |  | STRADB | 7.0748 |  | UBE2T | 5.3814 |  | TCF4 | 4.1327 |
| AQP3 | 3.7202 |  | SLC25A39 | 7.0407 |  | RRM2 | 5.3717 |  | IGKC | 4.0588 |
| CD2 | 3.6660 |  | BCL2L1 | 7.0329 |  | CDK1 | 5.3671 |  | ZFAT | 3.9642 |
| TRAC | 3.6028 |  | BNIP3L | 6.4764 |  | MKI67 | 5.3481 |  | PLD4 | 3.8824 |
| CD6 | 3.5786 |  | HMBS | 6.3504 |  | CCNA2 | 5.3280 |  | KCTD5 | 3.7746 |
| RORA | 3.5378 |  | TENT5C | 6.2702 |  | HMMR | 5.2997 |  | TRAF4 | 3.7568 |
| SPOCK2 | 3.4854 |  | GLRX5 | 6.0705 |  | CPOX | 5.2966 |  | VEGFB | 3.7281 |
| CD3G | 3.4738 |  | TSPAN5 | 6.0692 |  | CDKN3 | 5.2664 |  | SLC15A4 | 3.6138 |
| TRAT1 | 3.4165 |  | MPP1 | 6.0283 |  | CCNB2 | 5.2101 |  | PPP1R14B | 3.5862 |
| TBC1D4 | 3.3923 |  | PRDX2 | 5.9846 |  | TROAP | 5.1586 |  | NUDT17 | 3.5733 |
| MAL | 3.3424 |  | HAGH | 5.8578 |  | PLEKHH3 | 5.0784 |  | SIDT1 | 3.5093 |
| SYNE2 | 3.2065 |  | NFIX | 5.8550 |  | CENPF | 5.0681 |  | LDLRAD4 | 3.4983 |

| **C16**  **community** | **C16**  **logFC** |  | **C17**  **community** | **C17**  **logFC** |  | **C18**  **community** | **C18**  **logFC** |  | **C19**  **community** | **C19**  **logFC** |
| --- | --- | --- | --- | --- | --- | --- | --- | --- | --- | --- |
| NOCT | 5.5316 |  | BCL11B | 4.1996 |  | MZB1 | 9.2189 |  | IGHM | 7.8488 |
| MFSD2A | 4.6874 |  | SYNE2 | 4.0956 |  | DERL3 | 7.8834 |  | CD79B | 7.5862 |
| WDR91 | 4.6739 |  | GPRIN3 | 4.0139 |  | IGKC | 6.7163 |  | CCDC191 | 6.9764 |
| FUT4 | 4.3990 |  | FAM102A | 3.9498 |  | FKBP11 | 6.6534 |  | SPIB | 6.9307 |
| PKP4 | 4.3161 |  | AL356488.3 | 3.8486 |  | TXNDC5 | 6.5776 |  | NEIL1 | 6.7918 |
| AC083880.1 | 4.2563 |  | ZEB1 | 3.8314 |  | SEC11C | 6.3179 |  | CD79A | 5.9508 |
| AC211476.2 | 4.2189 |  | ARMCX4 | 3.7580 |  | IGHA1 | 6.2056 |  | CD72 | 5.7871 |
| CCNF | 4.2137 |  | LINC01772 | 3.6385 |  | PRDX4 | 5.4946 |  | MYO1C | 5.6303 |
| HNRNPLL | 4.1438 |  | SMAD7 | 3.6207 |  | CHPF | 5.3557 |  | MZB1 | 5.1535 |
| PAN3 | 4.0597 |  | PATJ | 3.6003 |  | CCR10 | 5.1573 |  | RUBCNL | 5.1473 |
| ERLIN1 | 4.0529 |  | AC139887.2 | 3.5990 |  | ITM2C | 4.8889 |  | CDC25B | 4.8105 |
| AL355075.4 | 4.0232 |  | SYTL2 | 3.5762 |  | HSP90B1 | 4.8366 |  | BCL7A | 4.7995 |
| AL391121.1 | 3.9439 |  | PIK3C2B | 3.5060 |  | TXNDC15 | 4.7599 |  | ABHD15 | 4.6401 |
| SENP3 | 3.9425 |  | CHD2 | 3.5002 |  | SSR4 | 4.7382 |  | IGKC | 4.5121 |
| SPEF2 | 3.9167 |  | TVP23C | 3.4888 |  | ZBP1 | 4.6885 |  | QRSL1 | 4.5115 |
| PHF13 | 3.9031 |  | GOLGA8B | 3.4800 |  | CD27 | 4.6690 |  | CD9 | 4.3988 |
| KDM5B | 3.8217 |  | AC091057.6 | 3.4757 |  | AQP3 | 4.6042 |  | CD22 | 4.3894 |
| BRCA2 | 3.7826 |  | L3MBTL1 | 3.4417 |  | PNOC | 4.5927 |  | ACSM3 | 4.3457 |
| LINC01970 | 3.7773 |  | ITK | 3.4345 |  | CD38 | 4.3752 |  | CXCR4 | 4.3445 |
| KCNQ1OT1 | 3.7621 |  | AL133245.1 | 3.3619 |  | GMPPB | 4.3394 |  | IGHD | 4.2576 |

| **C16**  **community** | **C16**  **logFC** |
| --- | --- |
| GBP1 | 4.4799 |
| DAB2 | 4.3467 |
| IFITM3 | 4.2617 |
| CLU | 4.0938 |
| GRAP2 | 3.9680 |
| F13A1 | 3.9502 |
| RGS18 | 3.6236 |
| ACRBP | 3.5539 |
| NR1H3 | 3.4943 |
| MS4A7 | 3.4486 |
| HMOX1 | 3.4057 |
| MAP3K7CL | 3.3818 |
| GBP5 | 3.3764 |
| PRKAR1B | 3.2876 |
| FTL | 3.2680 |
| FCER1G | 3.2652 |
| OAS1 | 3.2317 |
| CD68 | 3.1844 |
| PRDX1 | 3.1433 |
| TPM1 | 3.1368 |

***Note.*** Here, each label (e.g., C0, C1, …) represents a distinct community.
