# Supplementary material for: In-Silico discovery of Pediatric Acute-Myeloid-Leukemia (pAML) causing druggable molecular signatures highlighting their pathogenetic processes and therapeutic agents through single-cell RNA-Seq profile analysis
Source: PLoS One. 2025 Oct 31;20(10):e0335410. doi: 10.1371/journal.pone.0335410 (PMC12578151; doi:10.1371/journal.pone.0335410)
Supplement: S3 Table — (DOCX) [file pone.0335410.s010.docx]

## S3 Table. Cell type specificity scores and logFC values for evaluating top 20 marker genes' exclusivity in identifying specific cell types.

| **Basophils** | |  | **Dendritic cells** | |  | **Erythroid cells** | |  | **GdT cells** | |
| --- | --- | --- | --- | --- | --- | --- | --- | --- | --- | --- |
| **Genes** | **Scores** |  | **Genes** | **Scores** |  | **Genes** | **Scores** |  | **Genes** | **Scores** |
| ADAMTS10 | 13.4605 |  | CST3 | 72.6502 |  | SNCA | 76.5195 |  | GZMH | 46.1383 |
| SOCS2 | 51.5023 |  | CEBPD | 69.6930 |  | BLVRB | 84.6407 |  | FGFBP2 | 43.3508 |
| GOLGA8N | 25.4844 |  | TREM1 | 43.0019 |  | SLC25A37 | 80.8757 |  | GZMA | 72.4606 |
| AGRN | 10.3580 |  | EMP1 | 42.0021 |  | FAM210B | 78.9051 |  | KLRD1 | 49.2856 |
| NDRG2 | 18.0387 |  | GPAT3 | 24.6042 |  | HMBS | 66.2830 |  | GZMB | 42.2142 |
| OSM | 14.1829 |  | HMOX1 | 37.2394 |  | FECH | 70.6795 |  | GNLY | 53.1117 |
| COL9A2 | 25.5651 |  | CLEC7A | 49.3258 |  | TRIM58 | 51.1198 |  | PRF1 | 49.4565 |
| PRMT7 | 21.7094 |  | PHLDA1 | 20.6943 |  | BPGM | 49.3475 |  | CCL5 | 84.4645 |
| LCAT | 5.2081 |  | AREG | 35.0256 |  | DCAF12 | 42.6374 |  | KLRB1 | 52.6530 |
| PLD4 | 48.7098 |  | UPP1 | 59.8423 |  | CA2 | 57.3115 |  | GZMK | 38.7664 |
| AP1S3 | 11.9151 |  | TBC1D8 | 11.1171 |  | PRDX2 | 81.0071 |  | IL2RB | 25.3135 |
| CLIP2 | 14.7287 |  | IER3 | 34.8738 |  | STRADB | 60.2053 |  | NKG7 | 84.1020 |
| SLC15A2 | 5.4750 |  | SGK1 | 40.0589 |  | SLC25A39 | 76.6345 |  | CST7 | 76.1054 |
| RFLNB | 57.6677 |  | MIR22HG | 50.7851 |  | BCL2L1 | 57.0909 |  | CCL4 | 40.9798 |
| AZU1 | 55.3617 |  | LYZ | 48.6948 |  | ACSL6 | 17.8848 |  | SAMD3 | 29.3856 |
| ABCA2 | 11.1507 |  | SPHK1 | 13.6473 |  | CPOX | 41.7912 |  | GZMM | 65.3670 |
| LRFN4 | 7.8876 |  | SAT1 | 63.2403 |  | TSPAN5 | 33.2120 |  | KLRG1 | 27.5658 |
| KBTBD11 | 13.6412 |  | GPR183 | 39.9897 |  | GLRX5 | 78.0135 |  | MYOM2 | 7.7064 |
| SLC45A3 | 6.0127 |  | LINC01678 | 11.1055 |  | HAGH | 66.0818 |  | NCR3 | 22.6441 |
| KIAA0930 | 34.4696 |  | PIK3R5 | 26.3799 |  | SLC2A1 | 52.8397 |  | IL32 | 66.3351 |

| **Neutrophils** | |  | **Plasma cells** | |  | **Progenitor cells** | |  | **T-regs** | |
| --- | --- | --- | --- | --- | --- | --- | --- | --- | --- | --- |
| **Genes** | **Scores** |  | **Genes** | **Scores** |  | **Genes** | **Scores** |  | **Genes** | **Scores** |
| LYZ | 116.3666 |  | MZB1 | 30.3301 |  | EGFL7 | 128.6699 |  | RAD54L | 2.9604 |
| AC020656.1 | 72.0361 |  | DERL3 | 26.8352 |  | MPO | 104.2208 |  | CEP55 | 4.9111 |
| SMARCD3 | 30.1923 |  | IGKC | 20.9228 |  | PRSS57 | 126.2000 |  | UBE2C | 15.4939 |
| S100A11 | 102.5557 |  | FKBP11 | 27.7550 |  | FAM30A | 43.1989 |  | RECQL4 | 10.3029 |
| CST3 | 105.0853 |  | TXNDC5 | 18.3580 |  | FHL1 | 62.2974 |  | CENPM | 18.5821 |
| PTAFR | 41.1643 |  | SEC11C | 27.3425 |  | NPW | 64.9576 |  | MS4A3 | 12.8773 |
| FCER1G | 96.4682 |  | IGHA1 | 14.6416 |  | AMN | 32.7288 |  | PKMYT1 | 11.7147 |
| S100A6 | 111.9907 |  | PRDX4 | 26.2239 |  | CDCA7 | 53.7559 |  | CDCA5 | 9.9001 |
| TYROBP | 111.5864 |  | CHPF | 13.6582 |  | SPACA9 | 31.4083 |  | FOXM1 | 8.5795 |
| NCF1 | 81.3892 |  | CCR10 | 8.1400 |  | MIR181A1HG | 81.1350 |  | ASF1B | 9.6373 |
| LILRB3 | 37.0918 |  | ITM2C | 27.4820 |  | FSCN1 | 65.9243 |  | TYMS | 27.1968 |
| MNDA | 53.9400 |  | HSP90B1 | 27.7845 |  | JUP | 85.9174 |  | UHRF1 | 15.8783 |
| ITGAM | 39.8995 |  | TXNDC15 | 17.5925 |  | NPDC1 | 39.5922 |  | RNASEH2A | 13.3410 |
| CKAP4 | 27.2066 |  | SSR4 | 29.6802 |  | CD99 | 124.6379 |  | TK1 | 18.8436 |
| TYMP | 93.3244 |  | ZBP1 | 14.9294 |  | SERPINB1 | 119.9786 |  | KIF20A | 1.9316 |
| CLEC7A | 61.2495 |  | CD27 | 19.2166 |  | PDLIM1 | 96.7944 |  | GINS1 | 1.7645 |
| LINC00937 | 10.1555 |  | AQP3 | 16.2992 |  | LINC00539 | 25.0499 |  | MYBL2 | 12.9837 |
| MS4A6A | 78.2208 |  | PNOC | 10.1066 |  | ANKRD28 | 100.4805 |  | CDCA3 | 4.5164 |
| CTSS | 89.5773 |  | CD38 | 19.9774 |  | ITM2C | 106.3949 |  | PLK1 | 7.5006 |
| DMXL2 | 34.8240 |  | GMPPB | 12.3603 |  | TSC22D1 | 58.7625 |  | SAPCD2 | 5.1017 |

| **Naive B-cells** | |  | **Naive CD4 T-cells** | |  | **Non-classical monocytes** | |  | **Plasmacytoid dendritic cells** | |
| --- | --- | --- | --- | --- | --- | --- | --- | --- | --- | --- |
| **Genes** | **Scores** |  | **Genes** | **Scores** |  | **Genes** | **Scores** |  | **Genes** | **Scores** |
| MS4A1 | 63.6875 |  | LINC02446 | 31.5822 |  | FCGR3A | 71.0138 |  | TPM2 | 14.5930 |
| CD79A | 82.8227 |  | LEF1 | 53.9133 |  | CDKN1C | 47.2663 |  | DERL3 | 22.9517 |
| CD22 | 33.0287 |  | TRABD2A | 28.7846 |  | LILRA1 | 42.0509 |  | SPIB | 20.4303 |
| TNFRSF13C | 41.0956 |  | NELL2 | 25.7309 |  | SIGLEC10 | 40.2488 |  | C12orf75 | 33.4525 |
| IGHM | 71.0959 |  | CCR7 | 56.2277 |  | RRAS | 48.0041 |  | IRF4 | 15.0382 |
| IGHD | 50.9572 |  | CAMK4 | 44.5325 |  | LILRB1 | 49.5103 |  | AL096865.1 | 10.6709 |
| SPIB | 28.2476 |  | MAL | 43.5301 |  | FCER1G | 79.8612 |  | MZB1 | 26.2440 |
| BANK1 | 44.3663 |  | CD3E | 90.2682 |  | LILRB2 | 60.2775 |  | UGCG | 25.0270 |
| LINC00926 | 43.8518 |  | CD8B | 37.8540 |  | HMOX1 | 59.5246 |  | TCF4 | 28.2222 |
| PNOC | 14.0320 |  | IL7R | 67.1995 |  | S100A11 | 76.3993 |  | IGKC | 32.6434 |
| CD79B | 56.1579 |  | BCL11B | 40.2867 |  | LST1 | 78.3382 |  | ZFAT | 6.4430 |
| FCER2 | 31.2861 |  | CD3D | 81.5187 |  | CTSS | 76.1062 |  | PLD4 | 28.2620 |
| NEIL1 | 14.9669 |  | TCF7 | 59.1801 |  | AIF1 | 80.6260 |  | KCTD5 | 18.6862 |
| RALGPS2 | 31.8678 |  | CD3G | 58.0082 |  | BCL2A1 | 63.7396 |  | TRAF4 | 20.1875 |
| IGKC | 44.8824 |  | LTB | 87.9957 |  | MARCKS | 53.6595 |  | VEGFB | 16.9667 |
| RUBCNL | 21.6696 |  | TRAC | 53.3254 |  | PILRA | 54.3232 |  | SLC15A4 | 16.0785 |
| CD40 | 22.1979 |  | CD27 | 46.6093 |  | CEBPB | 68.0242 |  | PPP1R14B | 27.0701 |
| RAB30 | 16.6793 |  | ITK | 19.4314 |  | IFITM3 | 60.3444 |  | NUDT17 | 6.3246 |
| P2RX5 | 25.7984 |  | APBA2 | 17.9477 |  | C3AR1 | 24.5445 |  | SIDT1 | 9.1956 |
| SNX22 | 12.3589 |  | OXNAD1 | 32.3249 |  | MS4A7 | 49.6542 |  | LDLRAD4 | 14.3799 |
