# Supplementary material for: In-Silico discovery of Pediatric Acute-Myeloid-Leukemia (pAML) causing druggable molecular signatures highlighting their pathogenetic processes and therapeutic agents through single-cell RNA-Seq profile analysis
Source: PLoS One. 2025 Oct 31;20(10):e0335410. doi: 10.1371/journal.pone.0335410 (PMC12578151; doi:10.1371/journal.pone.0335410)
Supplement: S4 Table — (DOCX) [file pone.0335410.s011.docx]

## S4 Table. Context-specific cell-cell communication matrix between pAML and healthy samples.

|  | Basophils | Dendritic cells | Erythroid cells | GdT-cells | Neutrophils | Progenitor cells |
| --- | --- | --- | --- | --- | --- | --- |
| Basophils | 0.0472 | 0.0324 | 0.0699 | 0.0808 | 0.0408 | 0.0691 |
| Dendritic cells | 0.0513 | 0.0352 | 0.0760 | 0.0878 | 0.0443 | 0.0751 |
| Erythroid cells | 0.1039 | 0.0713 | 0.1538 | 0.1777 | 0.0896 | 0.1520 |
| GdT-cells | 0.0882 | 0.0605 | 0.1306 | 0.1509 | 0.0761 | 0.1290 |
| Neutrophils | 0.0485 | 0.0333 | 0.0719 | 0.0831 | 0.0419 | 0.0710 |
| Progenitor cells | 0.0449 | 0.0308 | 0.0664 | 0.0768 | 0.0387 | 0.0657 |
| T-regs | 0.0583 | 0.0400 | 0.0863 | 0.0998 | 0.0503 | 0.0853 |
| Naive B-cells | 0.0830 | 0.0570 | 0.1228 | 0.1420 | 0.0716 | 0.1214 |
| Naive CD4 T-cells | 0.0977 | 0.0671 | 0.1447 | 0.1672 | 0.0844 | 0.1430 |
| Non-classical monocytes | 0.0790 | 0.0542 | 0.1170 | 0.1352 | 0.0682 | 0.1156 |
| Plasmacytoid DCs | 0.0428 | 0.0294 | 0.0633 | 0.0732 | 0.0369 | 0.0626 |

|  | T-regs | Naive B-cells | Naive CD4 T-cells | Non-classical monocytes | Plasmacytoid DCs |
| --- | --- | --- | --- | --- | --- |
| Basophils | 0.0692 | 0.0331 | 0.0783 | 0.0782 | 0.0282 |
| Dendritic cells | 0.0751 | 0.0360 | 0.0850 | 0.0849 | 0.0306 |
| Erythroid cells | 0.1521 | 0.0728 | 0.1720 | 0.1719 | 0.0619 |
| GdT-cells | 0.1291 | 0.0618 | 0.1461 | 0.1460 | 0.0526 |
| Neutrophils | 0.0711 | 0.0340 | 0.0804 | 0.0803 | 0.0289 |
| Progenitor cells | 0.0657 | 0.0315 | 0.0743 | 0.0743 | 0.0267 |
| T-regs | 0.0854 | 0.0409 | 0.0966 | 0.0965 | 0.0348 |
| Naive B-cells | 0.1215 | 0.0582 | 0.1374 | 0.1373 | 0.0494 |
| Naive CD4 T-cells | 0.1431 | 0.0685 | 0.1619 | 0.1618 | 0.0583 |
| Non-classical monocytes | 0.1157 | 0.0554 | 0.1309 | 0.1308 | 0.0471 |
| Plasmacytoid DCs | 0.0626 | 0.0300 | 0.0708 | 0.0708 | 0.0255 |

***Note.*** Key sender and receiver cell types are highlighted in red.
