# Supplementary material for: In-Silico discovery of Pediatric Acute-Myeloid-Leukemia (pAML) causing druggable molecular signatures highlighting their pathogenetic processes and therapeutic agents through single-cell RNA-Seq profile analysis
Source: PLoS One. 2025 Oct 31;20(10):e0335410. doi: 10.1371/journal.pone.0335410 (PMC12578151; doi:10.1371/journal.pone.0335410)
Supplement: S5 Table — (DOCX) [file pone.0335410.s012.docx]

## S5 Table. List of upregulated and downregulated common DEGs (cDEGs) across key cell types.

| **Upregulated** |
| --- |
| BBC3, CUL4A, NRIP1, PSMA6, Z93241.1, SPCS3, UBE2V1, MAN2A1, MAPKAPK2, TOP1, MDM2, TXNDC11, SNX9, CKAP2, C17orf49, SARNP, UBALD2, NUP98, MIDN, NUFIP2, ERCC5, PDZD8, JUN, SEC14L1, RPRD1B, GABARAPL1, RCOR1, SUZ12, UBXN7, MAP3K8, TUBB2A, UBE2M, TCP11L2, FOSB, KAT6B, RIOK3, QKI, KDM5B, RBM27, FOXN2, MAP4K5, YRDC, GNAS, HERC5, ANKLE2, BTG3, SUPT6H, MT-ND6, CSRNP1, AFF1, DDIT3, UGCG, AL118516.1, USF3, RC3H2, ZBTB10, MATR3-1, HSPA13, ZCCHC2, TFE3, MRPL38, KLF6, UBE2S, ZNF326, RNF19A, RBBP6, HECTD4, CASC3, CBX4, TIMM23, FBXW7, CHD3, TUBA1A, RPL17, FAR1, ZFAND5, IER5, PLEKHM2, KLF10, FOS, CCDC71L, TIPARP, SKIL, MTHFD2L, PLCG2, ATP2B1-AS1, BTAF1, NFKBIZ, MAP2K2, PIM3, SERTAD1, USP12, CSNK2A2, POLR2J3, JUND, NR4A2, NFKBIE, SERTAD2, SREBF2, CSGALNACT2, NEU1, HIST1H1D, PTPN11, DYRK1A, JMY, NME2, BCL2L11, CDV3, MCL1, CLINT1, SLC7A5, STAM, EEF1G, NUMA1, ODC1, MXI1, PIK3CA, PTS, CNST, SDE2, H2AFJ, UHRF2, TXNRD1, MT-ATP8, MT-ND4L, WASHC2A, PELI1, PER1, C16orf87, TP53BP2, CNBD2, CD83, MEX3C, RBM15, ARL4A, ELL2, HAUS2, BRI3, MED13L, MXD4, NUDT4, ZFP91, RASGEF1B, GADD45B, MEF2D, FAM53C, SOD2, ZBTB43, RBM38, ISCA1, IGFLR1, HIST1H1E, PPP1R15A, LRRFIP2 |
| **Downregulated** |
| ING4, RAD9A, F8A1, HNRNPA1P48, AC004687.1, MTIF3, DYNC1I2, UGP2, NHLRC3, DENND2D, RBM14, NDUFB1, ZNF518A, IGKC, TCEAL8, RPA2, S1PR4, MITD1, PDHB, PSMA2, TRIM56, NFYB, KNOP1, ECI2, LUC7L3, PCMT1, MAP2K1, ATP5PO, AHSA1, PLRG1, IGHM, MRPL1, PHF14, GLRX, SMIM30, ICAM2, CNPY2, KRCC1, IGHA1, EMG1, DPY30, PYCR2, FTX, MRPL23 |
