# Supplementary material for: In-Silico discovery of Pediatric Acute-Myeloid-Leukemia (pAML) causing druggable molecular signatures highlighting their pathogenetic processes and therapeutic agents through single-cell RNA-Seq profile analysis
Source: PLoS One. 2025 Oct 31;20(10):e0335410. doi: 10.1371/journal.pone.0335410 (PMC12578151; doi:10.1371/journal.pone.0335410)
Supplement: S6 Table — (DOCX) [file pone.0335410.s013.docx]

## S6 Table. List of common key genes (cKGs) from the PPI network based on different topological measures.

| **Topological measures** | **Upregulated** | | | | | | | **Downregulated** |
| --- | --- | --- | --- | --- | --- | --- | --- | --- |
|  | **JUN** | **MDM2** | **FOS** | **SOD2** | **FBXW7** | **CHD3** | **MCL1** | **MAP2K1** |
| CLOSENESS | 111.3 | 103.5 | 94.8 | 92.6 | 103.6 | 90.4 | 91.8 | 89.2 |
| DEGREE | 58 | 45 | 35 | 29 | 43 | 24 | 27 | 22 |
| EPC | 60.2 | 57.9 | 57.0 | 53.1 | 56.9 | 52.4 | 54.0 | 48.4 |
| MNC | 57 | 45 | 35 | 27 | 40 | 22 | 27 | 21 |
| RADIALITY | 5.15 | 5.03 | 4.83 | 4.82 | 5.06 | 4.81 | 4.82 | 4.78 |
