# Supplementary material for: In-Silico discovery of Pediatric Acute-Myeloid-Leukemia (pAML) causing druggable molecular signatures highlighting their pathogenetic processes and therapeutic agents through single-cell RNA-Seq profile analysis
Source: PLoS One. 2025 Oct 31;20(10):e0335410. doi: 10.1371/journal.pone.0335410 (PMC12578151; doi:10.1371/journal.pone.0335410)
Supplement: S7 Table — (DOCX) [file pone.0335410.s014.docx]

## S7 Table. Association of cKGs with different diseases.

| **Disease Name** | ***p*-value** | **Associated KGs** |
| --- | --- | --- |
| Metastatic melanoma | 2.14E-09 | JUN; MAP2K1; FBXW7; MDM2; FOS; SOD2; MCL1 |
| **Acute lymphocytic leukemia** | 2.96E-09 | JUN; MAP2K1; FBXW7; MDM2; FOS; MCL1 |
| Squamous cell carcinoma of the head and neck | 3.64E-09 | JUN; FBXW7; MDM2; FOS; MCL1 |
| Pancreatic carcinoma | 5.74E-09 | JUN; MDM2; FOS; SOD2 |
| Malignant neoplasm of urinary bladder | 1.50E-08 | MAP2K1; FBXW7; MDM2; FOS; SOD2; MCL1 |
| Adenocarcinoma of lung (disorder) | 1.94E-08 | MAP2K1; FBXW7; MDM2; SOD2; MCL1 |
| Experimental Organism Basal Cell Carcinoma | 2.91E-08 | JUN; MAP2K1; FBXW7; MDM2; FOS; SOD2; MCL1 |
| Sarcoma | 3.28E-08 | JUN; MDM2; FOS; SOD2 |
| Lymphoma | 3.78E-08 | JUN; FBXW7; MDM2; FOS; SOD2 |
| Epithelial ovarian cancer | 4.25E-08 | JUN; MAP2K1; FBXW7; MDM2; FOS; SOD2; MCL1 |
| **Leukemogenesis** | 6.09E-08 | JUN; MAP2K1; FBXW7; MDM2; FOS; SOD2; MCL1 |
| **Leukemia, T-Cell** | 1.04E-07 | JUN; MDM2; FOS; SOD2; MCL1 |
| Neoplastic Cell Transformation | 1.21E-07 | JUN; MAP2K1; FBXW7; MDM2; FOS; CHD3; SOD2; MCL1 |
| **Myeloid Leukemia, Chronic** | 1.80E-07 | JUN; FBXW7; MDM2; FOS; MCL1 |
| Malignant neoplasm of kidney | 2.15E-07 | JUN; MAP2K1; MDM2; FOS; MCL1 |
| Adenocarcinoma | 2.46E-07 | JUN; MAP2K1; FBXW7; MDM2; FOS; SOD2; MCL1 |
| Classical Hodgkin's Lymphoma | 3.12E-07 | JUN; MAP2K1; FBXW7; MDM2; FOS; MCL1 |
| Mammary Neoplasms, Human | 3.95E-07 | JUN; FBXW7; MDM2; FOS; MCL1 |
| Malignant neoplasm of pancreas | 4.15E-07 | JUN; MDM2; FOS; SOD2 |
| Squamous cell carcinoma | 4.65E-07 | MAP2K1; FBXW7; MDM2; FOS; SOD2; MCL1 |
| Malignant neoplasm of mouth | 4.65E-07 | MAP2K1; FBXW7; MDM2; SOD2; MCL1 |
| Malignant neoplasm of prostate | 4.70E-07 | JUN; MAP2K1; FBXW7; MDM2; FOS; SOD2; MCL1 |
| **Adult T-Cell Lymphoma/Leukemia** | 5.04E-07 | JUN; MDM2; FOS; SOD2 |
| **Myeloid Leukemia** | 5.09E-07 | JUN; FBXW7; MDM2; FOS; SOD2 |
| **Childhood Acute Myeloid Leukemia** | 0.0400857 | MDM2 |
