# Supplementary material for: In-Silico discovery of Pediatric Acute-Myeloid-Leukemia (pAML) causing druggable molecular signatures highlighting their pathogenetic processes and therapeutic agents through single-cell RNA-Seq profile analysis
Source: PLoS One. 2025 Oct 31;20(10):e0335410. doi: 10.1371/journal.pone.0335410 (PMC12578151; doi:10.1371/journal.pone.0335410)
Supplement: S8 Table — (DOCX) [file pone.0335410.s015.docx]

## S8 Table. Collection of pAML-related candidate drug agents.

| **Sources** | **Drug Agents** |
| --- | --- |
| Kaspers and Creutzig., 2005 [1] | CYTARABINE, ANTHRACYCLINE, ETOPOSIDE |
| Kaspers and Zwaan., 2007 [2] | GEMTUZUMAB OZOGAMICIN, 2-CHLORO-DEOXYADENOSINE, LIPOSOMAL DAUNORUBICIN |
| Zwaan et al., 2000 [3] | GLUCOCORTICOIDS, ASPARAGINASE, ANTHRACYCLINES, DAUNORUBICIN, ACLARUBICIN, MITOXANTRONE, ETOPOSIDE, TENIPOSIDE, 6-MERCAPTOPURINE, AMSACRINE, 4-HYDROPEROXYIFOSFAMIDE, THIOTEPA, BUSULFAN, CISPLATIN, CARBOPLATIN, L-ASPARAGINASE, VINDESINE, PREDNISOLONE, DEXAMETHASONE |
| Rasche et al., 2018 [4] | 6-THIOGUANINE, PREDNISONE, VINCRISTINE, DOXORUBICIN, IDARUBICIN, INTRATHECAL CYTARABINE, CYCLOPHOSPHAMIDE |
| DrugBank [5] | DASATINIB, NILOTINIB, IVOSIDENIB, GILTERITINIB, IMATINIB, BUSULFAN, HEPATITIS B VACCINE (RECOMBINANT), CEDAZURIDINE, METHYLPREDNISONE, DIDANOSINE, PENTOSTATIN, TREOSULFAN, AZACITIDINE, ASPARAGINASE ERWINIA CHRYSANTHEMI, CLOFARABINE, FIBRINOGEN HUMAN, IMETELSTAT, TETRYZOLINE, SARGRAMOSTIM, CALCITRIOL, THIOSULFURIC ACID, PHYLLOQUINONE, CHOLECALCIFEROL, FLAVIN MONONUCLEOTIDE, ADALIMUMAB, ZALCITABINE, PLICAMYCIN, EDETIC ACID, SODIUM IODIDE, D-ALPHA-TOCOPHEROL ACETATE, VITAMIN A, GLYCEROL PHENYLBUTYRATE, BEROTRALSTAT, PALIVIZUMAB, TURPENTINE, SELENIOUS ACID, HAEMAGGLUTININ-STRAIN A(H3N2), TIAPROFENIC ACID, TETRADECYL HYDROGEN SULFATE (ESTER), SODIUM PHOSPHATE (DIBASIC, UNSPECIFIED FORM), EQUINE BOTULINUM NEUROTOXIN E IMMUNE FAB2, EQUINE BOTULINUM NEUROTOXIN C IMMUNE FAB2, RUPATADINE, DENOSUMAB, ERGOCALCIFEROL, ZINC, REVIPARIN, PHENYLPROPANOLAMINE, DEXBROMPHENIRAMINE, ETANERCEPT |
| DGIdb [6] | APIGENIN, VORICONAZOLE, RUTIN, INDOPROFEN, RESVERATROL, DEXAMETHASONE, GEFITINIB, ARTESUNATE, QUERCETIN, MYCOPHENOLATE, BORTEZOMIB, THIOGUANINE, MYCOPHENOLATE MOFETIL, EPOETIN ALFA, RIBAVIRIN, MAGNESIUM SULFATE ANHYDROUS, DEHYDRATED ALCOHOL, PACLITAXEL, URSODIOL, PILOCARPINE HYDROCHLORIDE, THROMBIN, BROMOCRIPTINE, NIMODIPINE, PHENOBARBITAL, BACLOFEN, REGORAFENIB, DOCETAXEL ANHYDROUS, VORINOSTAT, BELINOSTAT, PEMBROLIZUMAB, TEMSIROLIMUS, SIROLIMUS, IPILIMUMAB, CISPLATIN, NIVOLUMAB, METHOTREXATE, ASPARAGINASE, CYCLOPHOSPHAMIDE ANHYDROUS, VALPROIC ACID, DOXORUBICIN HYDROCHLORIDE, DAUNORUBICIN LIPOSOMAL, BRUCEANTIN, ATOMOXETINE HYDROCHLORIDE, MECHLORETHAMINE HYDROCHLORIDE, CINNARIZINE, QUINAPRIL HYDROCHLORIDE, TRIFLUPROMAZINE HYDROCHLORIDE, TROPISETRON, EFLORNITHINE, COLCHICINE, METHIMAZOLE, SERTRALINE HYDROCHLORIDE, GEMFIBROZIL, SODIUM SELENITE, CIPROFIBRATE, FENOFIBRATE MICRONIZED, DIPHENHYDRAMINE HYDROCHLORIDE, VINBLASTINE SULFATE, ARSENIC TRIOXIDE, CLOTRIMAZOLE, COPPER CHLORIDE, CLOFIBRATE, AZELASTINE HYDROCHLORIDE, BUPROPION HYDROCHLORIDE, AMINEPTIN, VEMURAFENIB, ERLOTINIB, HEXACHLOROPHENE, THIMEROSAL, ABIRATERONE ACETATE, ENZALUTAMIDE, VENETOCLAX, EPIRUBICIN, SULCONAZOLE NITRATE, PAROXETINE HYDROCHLORIDE (HEMIHYDRATE), IBRUTINIB, CABOZANTINIB S-MALATE, CAPIVASERTIB, TAMOXIFEN, MASOPROCOL, PYRITHIONE ZINC, ASTEMIZOLE, DOPAMINE, SUNITINIB, DECITABINE, TRIFLUPROMAZINE, BRIGATINIB, IRINOTECAN HYDROCHLORIDE, APOMORPHINE, IDELALISIB, TRAMETINIB DIMETHYL SULFOXIDE, BENDAMUSTINE, CLIOQUINOL, OLAPARIB, CLEMASTINE, SELUMETINIB, RITUXIMAB, CYTARABINE, OSIMERTINIB, RALOXIFENE HYDROCHLORIDE, PIMOZIDE, AMOXAPINE, VITAMIN K3, PERPHENAZINE, FULVESTRANT, PALBOCICLIB, GENISTEIN, ETHOPROPAZINE HYDROCHLORIDE, CRIZOTINIB, IODOQUINOL, LEVONORGESTREL, ZANUBRUTINIB, OXALIPLATIN, ECONAZOLE NITRATE, INOSITOL, ALECTINIB, PAZOPANIB, THIOTEPA, THIORIDAZINE, BENZALKONIUM CHLORIDE, DABRAFENIB, MELPHALAN, TAMOXIFEN CITRATE, NORTRIPTYLINE, LORLATINIB, RUXOLITINIB, ALEMTUZUMAB, SELINEXOR, SAPANISERTIB, PROPYLTHIOURACIL, CHLORAMBUCIL, TRIFLURIDINE, MITOMYCIN, OBINUTUZUMAB, LOPERAMIDE, MERCAPTOPURINE, ZINC CHLORIDE, AZATHIOPRINE, TRIAMTERENE, NICLOSAMIDE, SALMETEROL XINAFOATE, ENALAPRIL MALEATE, ALPELISIB, IFOSFAMIDE, TEMOZOLOMIDE, MITOXANTRONE, DIETHYLSTILBESTROL, MITOXANTRONE HYDROCHLORIDE, BEVACIZUMAB, PIRTOBRUTINIB, FLUPHENAZINE HYDROCHLORIDE, CHLORPROMAZINE, CETUXIMAB, NITAZOXANIDE, INTERFERON ALFA-2B, CLADRIBINE, CLOMIPRAMINE, GLUTARAL, ABEMACICLIB, TRABECTEDIN, DUVELISIB, FLUOROURACIL, IXAZOMIB, CAMPTOTHECIN, ACALABRUTINIB, CHLOROXINE, DOXORUBICIN LIPOSOME, FURAZOLIDONE, TOPOTECAN HYDROCHLORIDE, ENCORAFENIB, HYDRALAZINE, LENALIDOMIDE, BENZETHONIUM CHLORIDE, FENRETINIDE, PANITUMUMAB, GEMCITABINE, AXITINIB, PRAMLINTIDE, TRIFLUOPERAZINE, ANISINDIONE, CAPECITABINE, WARFARIN, SORAFENIB, ETOPOSIDE, CERITINIB, PROCHLORPERAZINE, MAPROTILINE, HALOPERIDOL DECANOATE, CARBOPLATIN, BITHIONOL, DAUNORUBICIN HYDROCHLORIDE, GRANISETRON, METHYLPREDNISOLONE, COBIMETINIB FUMARATE, METFORMIN, BINIMETINIB, DASATINIB ANHYDROUS, LEUCOVORIN CALCIUM, VANDETANIB, COBIMETINIB, SELUMETINIB SULFATE |

**References**

1. Kaspers GJL, Creutzig U. Pediatric acute myeloid leukemia: international progress and future directions. Leukemia 2005; 19:2025–2029

2. Kaspers GJL, Zwaan CM. Pediatric acute myeloid leukemia: towards high-quality cure of all patients. Haematologica 2007; 92:1519–1532

3. Zwaan CM, Kaspers G-JL, Pieters R, et al. Cellular drug resistance profiles in childhood acute myeloid leukemia: differences between FAB types and comparison with acute lymphoblastic leukemia. Blood, The Journal of the American Society of Hematology 2000; 96:2879–2886

4. Rasche M, Zimmermann M, Borschel L, et al. Successes and challenges in the treatment of pediatric acute myeloid leukemia: a retrospective analysis of the AML-BFM trials from 1987 to 2012. Leukemia 2018; 32:2167–2177

5. Wishart DS, Knox C, Guo AC, et al. DrugBank: a knowledgebase for drugs, drug actions and drug targets. Nucleic Acids Res 2008; 36:D901–D906

6. Cotto KC, Wagner AH, Feng Y-Y, et al. DGIdb 3.0: a redesign and expansion of the drug–gene interaction database. Nucleic Acids Res 2018; 46:D1068–D1073
