# Supplementary material for: In-Silico discovery of Pediatric Acute-Myeloid-Leukemia (pAML) causing druggable molecular signatures highlighting their pathogenetic processes and therapeutic agents through single-cell RNA-Seq profile analysis
Source: PLoS One. 2025 Oct 31;20(10):e0335410. doi: 10.1371/journal.pone.0335410 (PMC12578151; doi:10.1371/journal.pone.0335410)
Supplement: S9 Table — (DOCX) [file pone.0335410.s016.docx]

## S10 Table. Receptors identified as key genes (KGs) or hub genes (HGs) in the published literatures.

| **Articles** | **KGs or HGs** |
| --- | --- |
| Yan-Fang et al., 2012 [1] | FASLG, HDAC4, HDAC7 |
| Wang et al., 2022 [2] | ZC3H15, PPIG, DNTTIP2, UBE3A, PRPF40A, TMED5, GNL2 |
| Cai et al., 2021 [3] | FLT3, PF4, CD163, MRC1, CSF2RB, PPBP |
| Zhu et al., 2023 [4] | HOXA9, SORT1, SH3BP5 |
| He et al., 2025 [5] | CASP3, ELANE, HMOX1, CHUK, FLT1, JAK3, CTSL, AURKA |
| Sun et al., 2025 [6] | NEDD8 |

**References**

1. Yan-Fang T, Dong W, Li P, et al. Analyzing the gene expression profile of pediatric acute myeloid leukemia with real-time PCR arrays. Cancer Cell Int 2012; 12:1–12

2. Wang Q, Yue C, Liu Q, et al. Exploration of differentially expressed mRNAs and miRNAs for pediatric acute myeloid leukemia. Front Genet 2022; 13:865111

3. Cai D, Liang J, Cai X-D, et al. Identification of six hub genes and analysis of their correlation with drug sensitivity in acute myeloid leukemia through bioinformatics. Transl Cancer Res 2021; 10:126

4. Zhu H, Xu Y, Xia J, et al. Identification and analysis of methylation signature genes and association with immune infiltration in pediatric acute myeloid leukemia. J Cancer Res Clin Oncol 2023; 149:14965–14982

5. He M, Zhang X, Zhang J, et al. Evaluating the Role of Astragalus Polysaccharide in Modulating Immune Infiltration and Enhancing Prognostic Biomarkers in Pediatric Acute Myeloid Leukemia. Front Pharmacol 16:1538888

6. Sun J, Liu C, Yang G, et al. Targeting NEDD8 in pediatric acute myeloid leukemia: an integrated bioinformatics and experimental approach. Hematology 2025; 30:2478650
