# Supplementary material for: In-Silico discovery of Pediatric Acute-Myeloid-Leukemia (pAML) causing druggable molecular signatures highlighting their pathogenetic processes and therapeutic agents through single-cell RNA-Seq profile analysis
Source: PLoS One. 2025 Oct 31;20(10):e0335410. doi: 10.1371/journal.pone.0335410 (PMC12578151; doi:10.1371/journal.pone.0335410)
Supplement: S11 Table — (DOCX) [file pone.0335410.s018.docx]

## S11 Table. Docking scores (binding affinities, kcal/mol) between the proposed drug candidates and 27 different receptors identified as key genes (KGs) or hub genes (HGs) in the published literatures.

|  | IMATINIB | IRINOTECAN HYDROCHLORIDE | IBRUTINIB |
| --- | --- | --- | --- |
| JAK3 | -9.6 | -9.2 | -10.5 |
| FLT1 | -9.6 | -9.4 | -9.8 |
| AURKA | -9.8 | -8.5 | -10.4 |
| HDAC7 | -8.8 | -10.6 | -9.1 |
| FLT3 | -9.4 | -8.7 | -9.1 |
| SORT1 | -8.8 | -8.6 | -9.6 |
| CASP3 | -8.3 | -9.5 | -8.8 |
| CTSL | -9.1 | -9.4 | -8.1 |
| GNL2 | -8.3 | -8.9 | -8.4 |
| HMOX1 | -8.4 | -8.6 | -8.4 |
| CD163 | -8.7 | -8.5 | -8 |
| ZC3H15 | -8.6 | -7.5 | -8.8 |
| PPIG | -8.6 | -8 | -8.2 |
| ELANE | -7.8 | -9.1 | -7.9 |
| CHUK | -7.8 | -8 | -8.6 |
| SH3BP5 | -8.1 | -8.4 | -7.8 |
| HOXA9 | -8.4 | -7.8 | -7.5 |
| PRPF40A | -7.7 | -8 | -7.9 |
| MRC1 | -8 | -7.9 | -7.5 |
| TMED5 | -7.6 | -7.5 | -8.2 |
| DNTTIP2 | -7.9 | -7.5 | -7.4 |
| NEDD8 | -8.2 | -7.4 | -6.9 |
| FASLG | -7.4 | -7.6 | -7.2 |
| CSF2RB | -7.8 | -7.5 | -6.8 |
| UBE3A | -7.3 | -7.3 | -6.9 |
| HDAC4 | -7.1 | -7.8 | -6.5 |
| PF4 | -7.2 | -6.9 | -7.1 |

*Note.* Here, the rows represent the drugs and the columns represent the receptors.
