# Supplementary material for: In-Silico discovery of Pediatric Acute-Myeloid-Leukemia (pAML) causing druggable molecular signatures highlighting their pathogenetic processes and therapeutic agents through single-cell RNA-Seq profile analysis
Source: PLoS One. 2025 Oct 31;20(10):e0335410. doi: 10.1371/journal.pone.0335410 (PMC12578151; doi:10.1371/journal.pone.0335410)
Supplement: S12 Table — (DOCX) [file pone.0335410.s019.docx]

## S12 Table. The identifiers of the Decoy molecules generated for IMATINIB, IBRUTINIB, and IRINOTECAN HYDROCLORIDE.

| **Target Receptors** | **Generated Decoys** |
| --- | --- |
| IMATINIB | C00867116, C01131634, C01259799, C03012211, C03630129, C06195338, C08812047, C09090169, C09193922, C09562338, C09694620, C11695908, C11841231, C11881999, C12771737, C12844353, C13131503, C13501330, C13501494, C13501496, C13506344, C13640561, C19851826, C21422517, C22796381, C25394021, C26681013, C27550417, C27550615, C27550674, C33790282, C36601958, C38547668, C38659142, C38935258, C48990333, C59489486, C63135270, C63187736, C63270971, C63383479, C63383509, C63789201, C64713315, C64794519, C65074714, C70972830, C71892198, C98262552, C98306154 |
| IBRUTINIB | C00703775, C03354294, C08602082, C08887432, C09057114, C09260569, C09298493, C09320637, C09432357, C09522041, C09660394, C09726942, C09783344, C09809526, C09882684, C11317196, C12007243, C12385025, C13005247, C13069880, C13496596, C16534130, C16554179, C16667100, C20555829, C24842049, C24842053, C30657066, C32071292, C32079425, C32668003, C32774462, C32985235, C33020349, C34984026, C35427741, C40823668, C48344727, C48368334, C48986703, C49410753, C58406496, C64891839, C65307884, C71802600, C77972066, C78777345, C96991439, C97549522, C98352117 |
| IRINOTECAN HYDROCHLORIDE | C00702977, C00905811, C01317230, C02668267, C03915436, C04790706, C05396040, C06077710, C06177994, C06195048, C08413305, C08413474, C08425664, C08453821, C10249069, C10249466, C16449575, C17434052, C20510182, C20571932, C33356553, C34676828, C34830454, C35926564, C35926622, C36074431, C36215083, C37089028, C38150058, C42919869, C59331784, C63136941, C63137672, C63138964, C63140215, C63362198, C63362542, C63375337, C70665426, C70670136, C70707973, C70708247, C70708590, C71389016, C77291189, C96019747, C98006372, C98012350, C98044502, C98232201 |
