# Supplementary material for: In-Silico discovery of Pediatric Acute-Myeloid-Leukemia (pAML) causing druggable molecular signatures highlighting their pathogenetic processes and therapeutic agents through single-cell RNA-Seq profile analysis
Source: PLoS One. 2025 Oct 31;20(10):e0335410. doi: 10.1371/journal.pone.0335410 (PMC12578151; doi:10.1371/journal.pone.0335410)
Supplement: S13 Table — (DOCX) [file pone.0335410.s020.docx]

## S13 Table. The average binding affinity scores (BASs) of the decoy molecules and the BAS of suggested drug candidates in kcal/mol with the target receptors.

| **Target**  **Receptors** | **Average BAS with Negative Control (Decoy)** | **BAS with Repurposed Drug Molecules** | **Drug Molecules** |
| --- | --- | --- | --- |
| **MAP2K1** | -6.6 | -10.7 | **IMATINIB** |
|  | -7.0 | -10.6 | **IBRUTINIB** |
|  | -6.1 | -10.8 | **IRINOTECAN HYDROCHLORIDE** |
| **CHD3** | -6.0 | -8.6 | **IMATINIB** |
|  | -6.4 | -8.9 | **IBRUTINIB** |
|  | -6.8 | -9.4 | **IRINOTECAN HYDROCHLORIDE** |
| **MCL1** | -6.2 | -10.2 | **IMATINIB** |
|  | -6.5 | -9.6 | **IBRUTINIB** |
|  | -6.3 | -9.9 | **IRINOTECAN HYDROCHLORIDE** |
| **TP53** | -5.1 | -8.6 | **IMATINIB** |
|  | -5.5 | -8.3 | **IBRUTINIB** |
|  | -5.6 | -9.3 | **IRINOTECAN HYDROCHLORIDE** |
| **SOD2** | -5.0 | -8.4 | **IMATINIB** |
|  | -5.4 | -8.1 | **IBRUTINIB** |
|  | -5.4 | -8.2 | **IRINOTECAN HYDROCHLORIDE** |
| **GATA2** | -5.1 | -7.9 | **IMATINIB** |
|  | -5.4 | -8.7 | **IBRUTINIB** |
|  | -5.8 | -7.5 | **IRINOTECAN HYDROCHLORIDE** |
| **RELA** | -4.6 | -7.4 | **IMATINIB** |
|  | -5.0 | -7.2 | **IBRUTINIB** |
|  | -5.1 | -7.2 | **IRINOTECAN HYDROCHLORIDE** |
| **FBXW7** | -4.7 | -7.3 | **IMATINIB** |
|  | -5.0 | -7.4 | **IBRUTINIB** |
|  | -5.2 | -7.8 | **IRINOTECAN HYDROCHLORIDE** |
| **FOXC1** | -4.3 | -7.5 | **IMATINIB** |
|  | -4.4 | -7.0 | **IBRUTINIB** |
|  | -4.9 | -8.4 | **IRINOTECAN HYDROCHLORIDE** |
| **MDM2** | -4.5 | -6.9 | **IMATINIB** |
|  | -4.9 | -6.8 | **IBRUTINIB** |
|  | -4.9 | -7.7 | **IRINOTECAN HYDROCHLORIDE** |
| **JUN** | -3.8 | -6.4 | **IMATINIB** |
|  | -4.0 | -6.3 | **IBRUTINIB** |
|  | -4.4 | -6.4 | **IRINOTECAN HYDROCHLORIDE** |
| **FOS** | -4.5 | -7.0 | **IMATINIB** |
|  | -4.1 | -6.6 | **IBRUTINIB** |
|  | -4.2 | -7.5 | **IRINOTECAN HYDROCHLORIDE** |
