# Supplementary material for: In-Silico discovery of Pediatric Acute-Myeloid-Leukemia (pAML) causing druggable molecular signatures highlighting their pathogenetic processes and therapeutic agents through single-cell RNA-Seq profile analysis
Source: PLoS One. 2025 Oct 31;20(10):e0335410. doi: 10.1371/journal.pone.0335410 (PMC12578151; doi:10.1371/journal.pone.0335410)
Supplement: S14 Table — (DOCX) [file pone.0335410.s021.docx]

## S14 Table. Determined FMO energies along with their corresponding physicochemical descriptors, such as chemical hardness, electronegativity, softness, chemical potential, and global electrophilicity index

| **Parameter** | **IRINOTECAN HYDROCHLORIDE** | **IMATINIB** | **IBRUTINIB** |
| --- | --- | --- | --- |
| LUMO energy (E_LUMO_) | -5.5909 | -5.239 | -4.9794 |
| HOMO energy (E_HOMO_) | -8.303 | -8.0135 | -7.8241 |
| Energy gap (∆E = E_LUMO_ *-* E_HOMO_) | 2.7122 | 2.7745 | 2.8447 |
| Ionization potential (I) | 8.303 | 8.0135 | 7.8241 |
| Electron affinity (A) | 5.5909 | 5.239 | 4.9794 |
| Chemical hardness (η) | 2.712159 | 2.712159 | 2.844678 |
| Softness (σ) | 0.36871 | 0.36871 | 0.351534 |
| Electro-negativity (χ) | 6.9469 | 6.9469 | 6.4018 |
| Chemical potential (µ) | -6.94693 | -6.62624 | -6.40175 |
| electrophilicity (ω) | 8.896943 | 7.912695 | 7.203347 |
