# Supplementary material for: In-Silico discovery of Pediatric Acute-Myeloid-Leukemia (pAML) causing druggable molecular signatures highlighting their pathogenetic processes and therapeutic agents through single-cell RNA-Seq profile analysis
Source: PLoS One. 2025 Oct 31;20(10):e0335410. doi: 10.1371/journal.pone.0335410 (PMC12578151; doi:10.1371/journal.pone.0335410)
Supplement: S1 Fig — (A) Sample-specific clustering observed before batch correction; (B) Uniform cell distribution achieved after batch correction. Here, each dot represents a cell, and each color represents a distinct sample. (DOCX) [file pone.0335410.s022.docx]

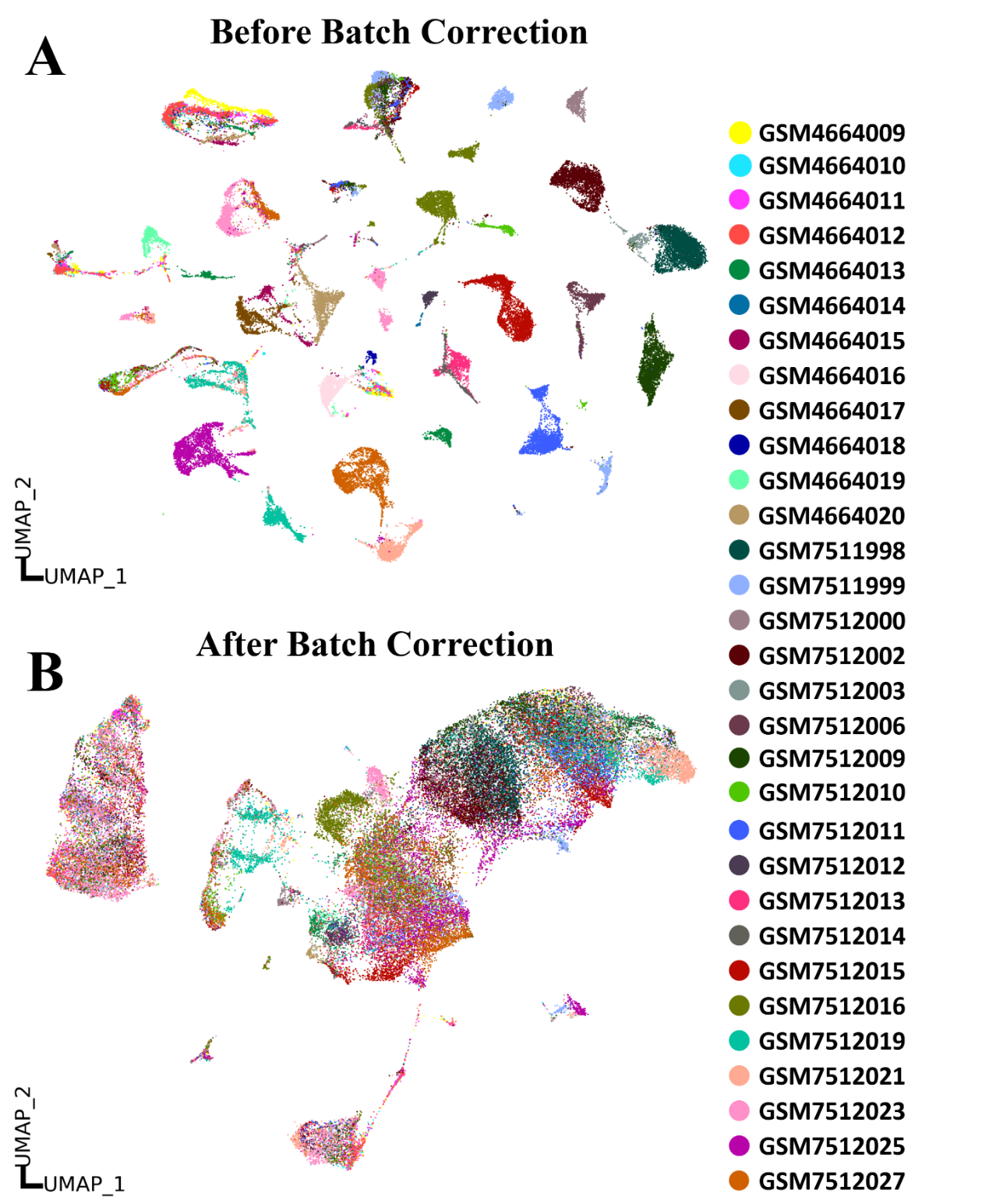


**S1 Figure.** UMAP plot of scRNA-seq data. **(A)** Sample-specific clustering observed before batch correction; **(B)** Uniform cell distribution achieved after batch correction. Here, each dot represents a cell, and each color represents a distinct sample.
