# Supplementary figures and images for: In-Silico discovery of Pediatric Acute-Myeloid-Leukemia (pAML) causing druggable molecular signatures highlighting their pathogenetic processes and therapeutic agents through single-cell RNA-Seq profile analysis
Source: PLoS One. 2025 Oct 31;20(10):e0335410. doi: 10.1371/journal.pone.0335410 (PMC12578151; doi:10.1371/journal.pone.0335410)

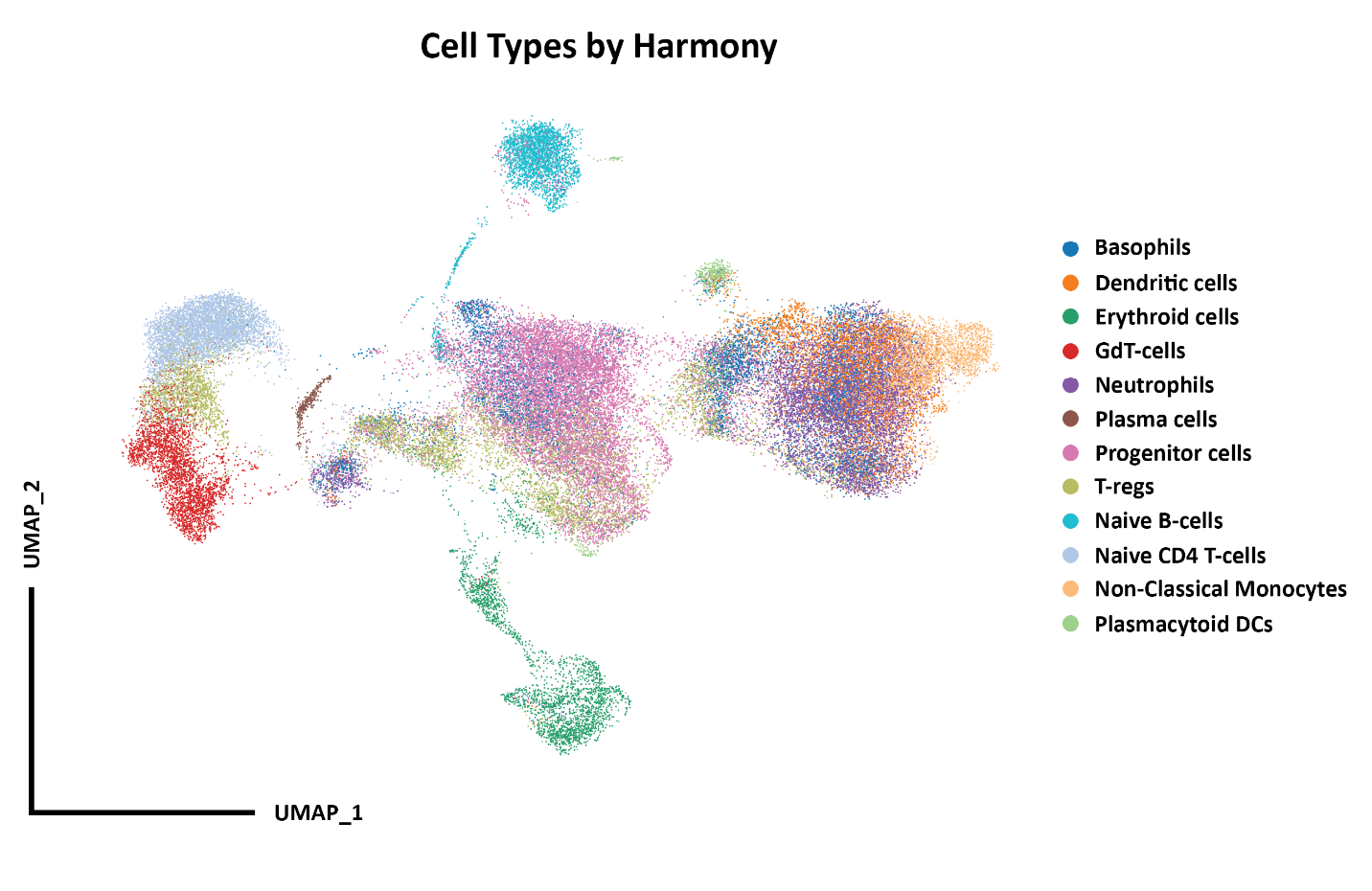


**S2 Figure.** UMAP plot of cell types clustering for Harmony integration.

Supplement: S2 Fig — (DOCX) [file pone.0335410.s023.docx]

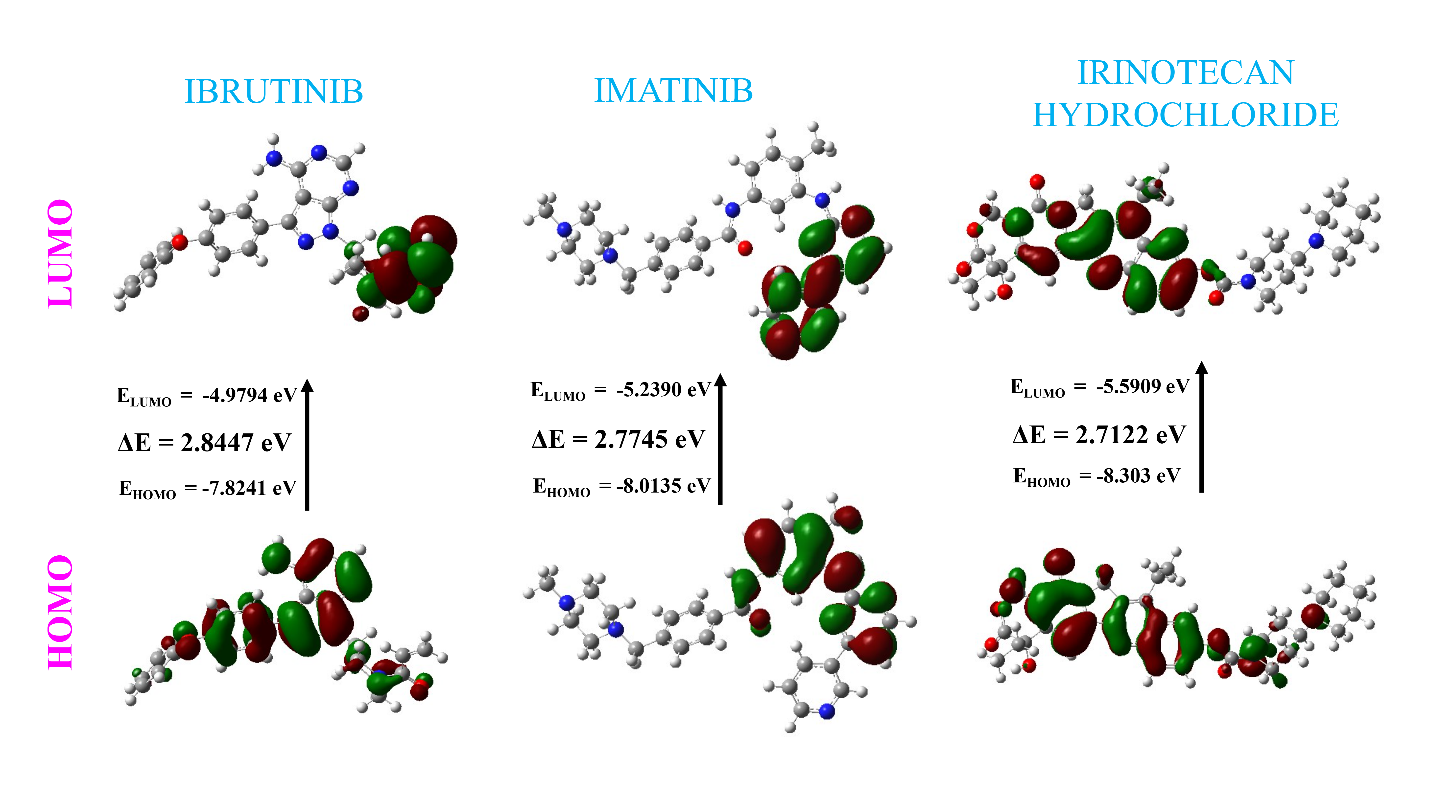


**S5 Figure.** The HOMO and LUMO orbitals of the proposed drug molecules.

Supplement: S5 Fig — (DOCX) [file pone.0335410.s026.docx]
