# Supplementary material for: In-Silico discovery of Pediatric Acute-Myeloid-Leukemia (pAML) causing druggable molecular signatures highlighting their pathogenetic processes and therapeutic agents through single-cell RNA-Seq profile analysis
Source: PLoS One. 2025 Oct 31;20(10):e0335410. doi: 10.1371/journal.pone.0335410 (PMC12578151; doi:10.1371/journal.pone.0335410)
Supplement: S3 Fig — Rows and top column annotations represent cell types, while the lower columns correspond to their associated marker genes. Color intensity indicates column-scaled expression levels, with darker shades reflecting higher expression. (DOCX) [file pone.0335410.s024.docx]

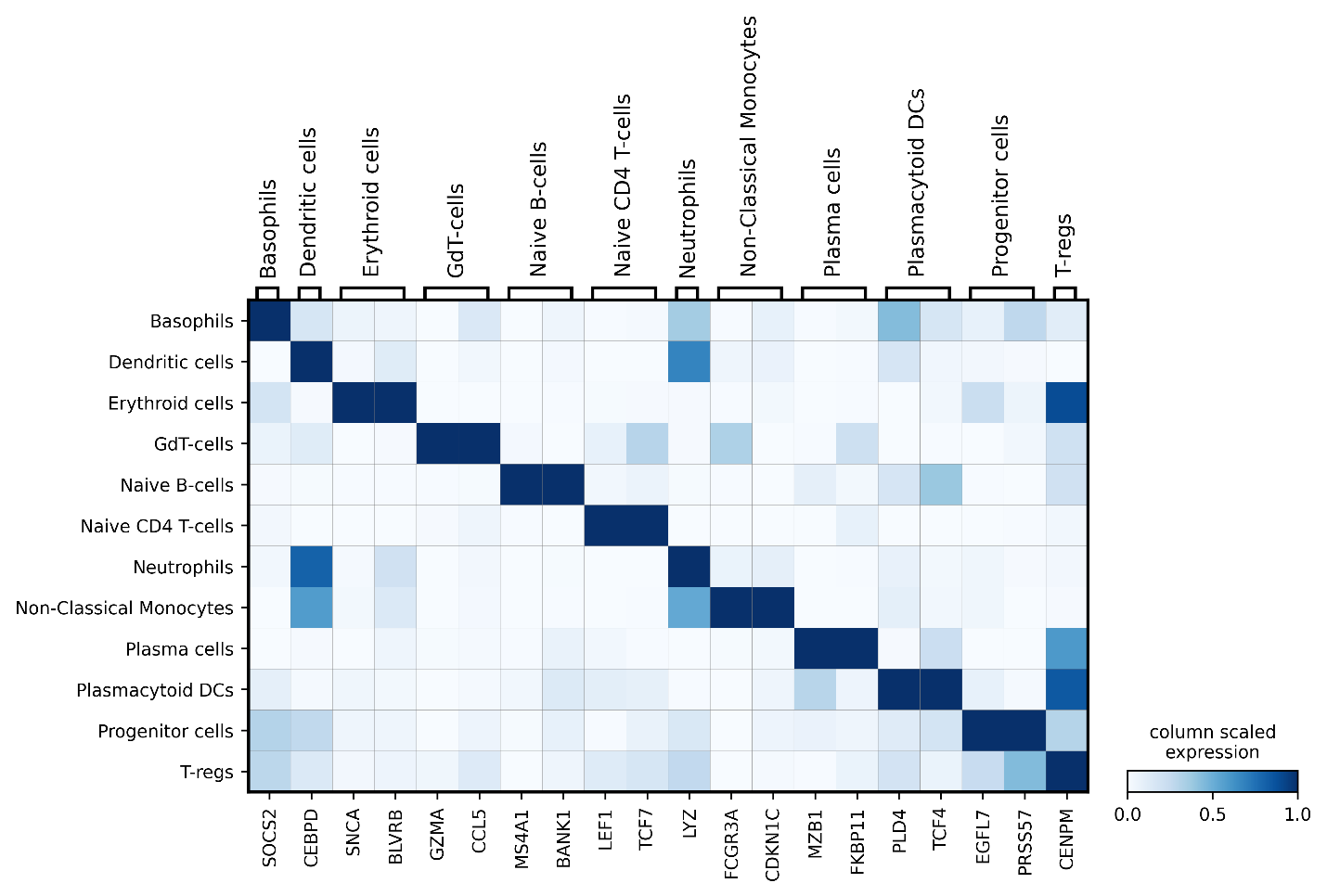


**S3 Figure.** Matrix plot displaying marker genes of corresponding cell types. Rows and top column annotations represent cell types, while the lower columns correspond to their associated marker genes. Color intensity indicates column-scaled expression levels, with darker shades reflecting higher expression.
