# Supplementary material for: In-Silico discovery of Pediatric Acute-Myeloid-Leukemia (pAML) causing druggable molecular signatures highlighting their pathogenetic processes and therapeutic agents through single-cell RNA-Seq profile analysis
Source: PLoS One. 2025 Oct 31;20(10):e0335410. doi: 10.1371/journal.pone.0335410 (PMC12578151; doi:10.1371/journal.pone.0335410)
Supplement: S4 Fig — (A) Venn diagram of upregulated cDEGs among six key cell types that shows 154 upregulated cDEGs and (B) Venn diagram of downregulated cDEGs among six key cell types that shows 44 downregulated cDEGs. (DOCX) [file pone.0335410.s025.docx]

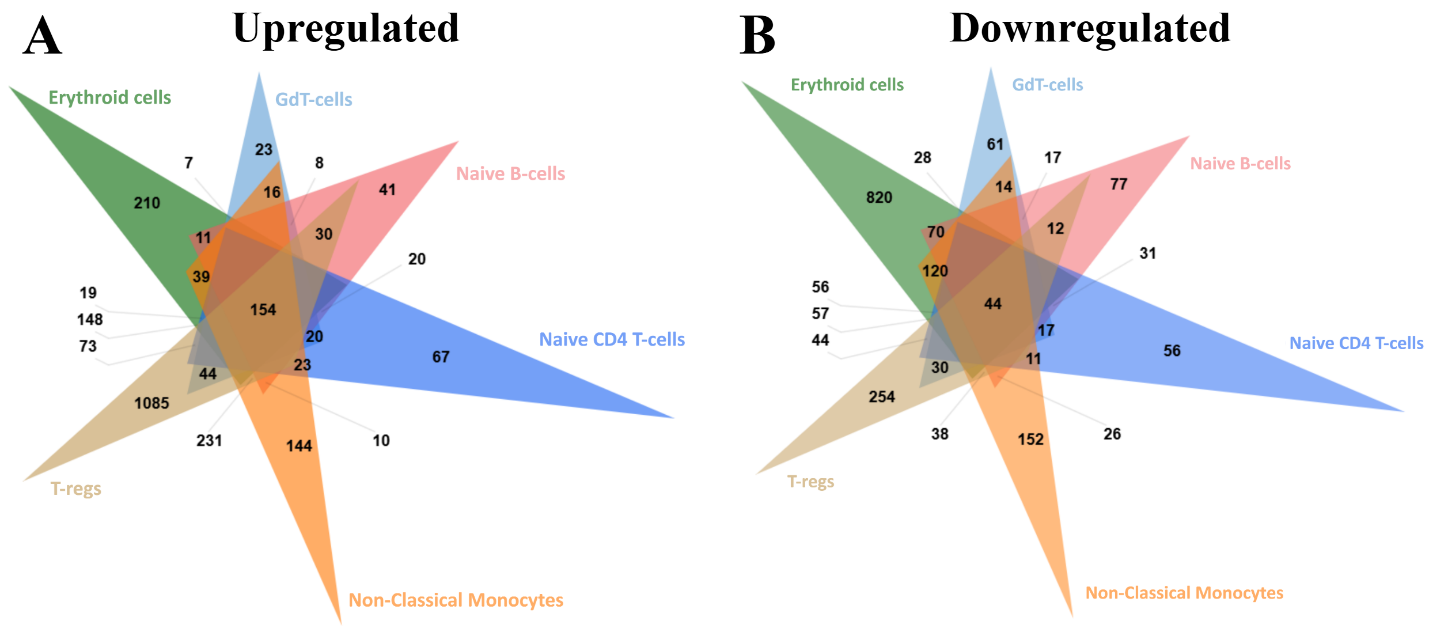


**S4 Figure.** Venn diagram of cDEGs among six key cell types. (**A**) Venn diagram of upregulated cDEGs among six key cell types that shows 154 upregulated cDEGs and (**B**) Venn diagram of downregulated cDEGs among six key cell types that shows 44 downregulated cDEGs.
